# F_1_F_O_ ATP synthase molecular motor mechanisms

**DOI:** 10.3389/fmicb.2022.965620

**Published:** 2022-08-23

**Authors:** Wayne D. Frasch, Zain A. Bukhari, Seiga Yanagisawa

**Affiliations:** School of Life Sciences, Arizona State University, Tempe, AZ, United States

**Keywords:** F_1_F_o_ ATP synthase, F_1_ ATPase, single-molecule studies, rotary molecular motor, torque

## Abstract

The F-ATP synthase, consisting of F_1_ and F_O_ motors connected by a central rotor and the stators, is the enzyme responsible for synthesizing the majority of ATP in all organisms. The F_1_ (αβ)_3_ ring stator contains three catalytic sites. Single-molecule F_1_ rotation studies revealed that ATP hydrolysis at each catalytic site (0°) precedes a power-stroke that rotates subunit-γ 120° with angular velocities that vary with rotational position. Catalytic site conformations vary relative to subunit-γ position (β_E_, empty; β_D_, ADP bound; β_T_, ATP-bound). During a power stroke, β_E_ binds ATP (0°–60°) and β_D_ releases ADP (60°–120°). Årrhenius analysis of the power stroke revealed that elastic energy powers rotation *via* unwinding the γ-subunit coiled-coil. Energy from ATP binding at 34° closes β_E_ upon subunit-γ to drive rotation to 120° and forcing the subunit-γ to exchange its tether from β_E_ to β_D_, which changes catalytic site conformations. In F_1_F_O_, the membrane-bound F_O_ complex contains a ring of c-subunits that is attached to subunit-γ. This c-ring rotates relative to the subunit-a stator in response to transmembrane proton flow driven by a pH gradient, which drives subunit-γ rotation in the opposite direction to force ATP synthesis in F_1_. Single-molecule studies of F_1_F_O_ embedded in lipid bilayer nanodisks showed that the c-ring transiently stopped F_1_-ATPase-driven rotation every 36° (at each c-subunit in the c_10_-ring of *E. coli* F_1_F_O_) and was able to rotate 11° in the direction of ATP synthesis. Protonation and deprotonation of the conserved carboxyl group on each c-subunit is facilitated by separate groups of subunit-a residues, which were determined to have different pKa’s. Mutations of any of any residue from either group changed both pKa values, which changed the occurrence of the 11° rotation proportionately. This supports a Grotthuss mechanism for proton translocation and indicates that proton translocation occurs during the 11° steps. This is consistent with a mechanism in which each 36° of rotation the c-ring during ATP synthesis involves a proton translocation-dependent 11° rotation of the c-ring, followed by a 25° rotation driven by electrostatic interaction of the negatively charged unprotonated carboxyl group to the positively charged essential arginine in subunit-a.

## The F-type, A-type, and V-type family of rotary molecular motors

**Figure 1:**
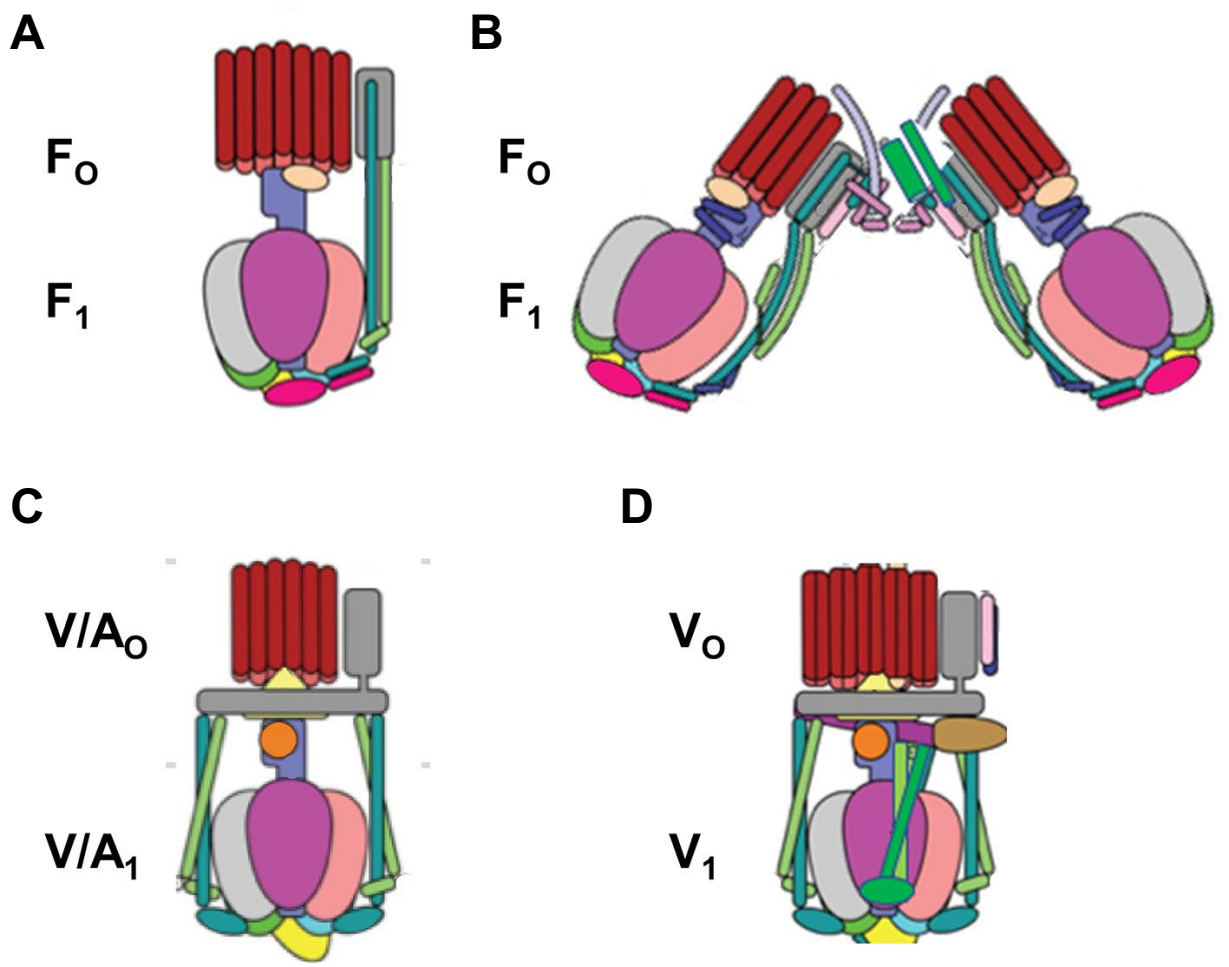
Structural variations among the family of rotary ATP synthases and ATPases that are coupled to transmembrane proton, or rarely sodium, gradients. **(A)** F-type ATP synthases in bacteria and plant chloroplasts (pdb-IDs 6OQR and 6FKF). **(B)** F-type ATP synthases in mitochondria (pdb-ID 6B8H). **(C)** V-type ATPases in some bacteria such as *E. hirae* and V/A-type ATP synthases in archaebacteria (pdb-ID 6R0Z) **(D)** V-type ATPases in vacuoles (pdb-ID 3J9V). V-type motors are incapable of synthesizing ATP and are used to pump protons to create a transmembrane pH gradient.

The F_1_F_O_ ATP synthase (Figure 1), which is found in all animals, plants, and eubacteria, provides the largest source of ATP that fuels most cellular processes (Spetzler et al., 2012; Kühlbrandt, 2019). The F_1_F_o_ complexes use a non-equilibrium proton gradient (or Na^+^ gradient in some organisms) to drive the ATP/ADP•Pi concentration ratio far from equilibrium. Most cellular processes use ATP as an energy source, which returns the concentration ratio toward equilibrium upon hydrolysis. Consistent with its vital role to life on earth, evolutionary variations of F_1_F_O_ are now known that enable life forms to survive in a wide variety of environmental conditions. Complete structures of F_1_F_O_ have now been determined from a variety of organisms. Although the identity of rotor and stator subunits in each motor is largely the same, variations in activity-altering loop regions are present in some subunits, and F_1_F_O_ from some species contain additional unique subunits. For example, additional subunits in mammalian facilitate the formation of F_1_F_O_ dimers. The F-type ATP synthases are also members of an extended family of rotary motors that include archaeal A-type (A_1_A_O_) ATP-synthases, prokaryotic A/V-type ATP synthases, as well as prokaryotic and eukaryotic vacuolar V-type ATPases (V_1_V_O_) that hydrolyze ATP to generate a transmembrane proton gradient. All share a common core of structural features that are embodied in F_1_F_O_ from *E. coli* (*Ec*F_1_F_O_).

**Figure 2:**
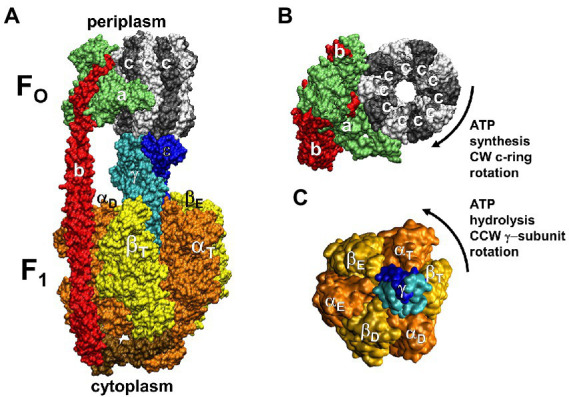
Subunit composition of *Ec*F_1_F_O_ (pdb-ID 6OQR) from the side **(A)**, of F_O_ from the periplasm **(B)**, and of solubilized F_1_ **(C)** from the periplasm (pdb-ID 3OAA). Peripheral stalk, b-subunits; central stalk, γ– and ε–subunits; rotor subunits, γ–, ε–subunits and the c-ring; catalytic sites, β-subunits; proton-translocating half- channels, subunit-a. Each c-subunit carries one proton between subunit-a half-channels during rotation.

The F_1_F_O_ ATP synthases are comprised of two rotary molecular motors (the F_1_ and F_O_ complexes) that are attached by their rotors and their stators (Figure 2). The F_O_ motor, which is embedded in bioenergetic membranes, uses a non-equilibrium transmembrane chemiosmotic proton gradient (or a Na^+^ gradient) known as a proton-motive force (pmf), to power clockwise (CW) rotation of its ring of c-subunits relative to stator subunits a and b as viewed from the *E. coli* periplasm. These subunits contribute to the peripheral stalk bound to one side of the F_1_ (αβ)_3_-subunit ring, which collectively serve as the stators of both motors. The F_1_ motor is a peripheral protein complex that in eukaryotes is exposed to the mitochondrial matrix or the chloroplast stroma. In bacteria such as *E. coli*, F_1_ is exposed to the cytoplasm. Extending through the core of the (αβ)_3_-ring, the F_1_ subunit-γ forms a central stalk that, with subunit-ε, docks to the c-ring of F_O_. The F_1_ motor is capable of catalyzing ATP hydrolysis-driven CCW rotation that pumps protons across the membrane to create a proton gradient. However, a variety of regulatory mechanisms have evolved in different organisms to minimize hydrolysis, which can be a wasteful process.

The F_1_ complex can be purified from the membrane and studied as an ATPase independently from F_O_. Each αβ–heterodimer comprises a catalytic site where most of the catalytic residues reside on subunit-β. Three additional non-catalytic binding sites are present at the alternate αβ interfaces where residues on subunit-α contribute to nucleotide binding.

### The binding-change/alternating site hypothesis

The alternating site (or binding-change) mechanism of ATP synthesis was proposed to explain the results of experiments that measured isotope exchange among the substrates (ADP and Pi) and products (ATP and H_2_O) as a function of nucleotide concentration (Boyer and Kohlbrenner, 1981; O’Neal and Boyer, 1984). This mechanism posited that the binding of ADP and Pi at one catalytic site induces a conformation change of all three catalytic sites to create an environment in which the equilibrium constant of tightly bound substrates and products at a second site approaches unity. It was estimated that interconversion of substrates and products at this latter site can occur up to 400 times prior to product release and suggested that energy from the pmf was required to induce a conformational change that enabled the release of product ATP from one site while ATP synthesis at another site could occur without significant energy input. It was proposed that this was possible because, at any moment, the three catalytic sites were in different conformations and that rotation of subunit-γ forces successive conformational changes of each site.

#### Evidence of coordinated, sequential catalytic site conformations changes

Early evidence supporting the binding-change hypothesis was obtained when VO^2+^ was used as a functional surrogate for Mg^2+^ in F_1_ purified from *Spinacia oleracea* chloroplasts (*So*CF_1_) (Frasch, 2000a,b). Nucleotides bind to the F_1_ catalytic and non-catalytic sites as a complex with Mg^2+^ (Abrahams et al., 1994), which serves as a cofactor for ATP hydrolysis. The VO^2+^ studies revealed that the conformations of the three catalytic sites are staggered, and that they all change in a concerted, sequential manner through the three conformations (when ATP binds to the lowest affinity site). Methods had been established to replace the Mg^2+^-nucleotide bound specifically to each of three sites in the (αβ)_3_-ring of *So*CF_1_ (Bruist and Hammes, 1981). Catalytic Site-3 can bind Mg-ATP or Mg-ADP with μM affinity, which can be depleted *via* gel filtration. Non-catalytic Site-2 binds only Mg-ATP that will not dissociate even after extensive catalytic turnover, but can be depleted as the result of partial unfolding *via* ammonium sulfate precipitation in the presence of EDTA. Site-1 contains tightly bound Mg-ADP that is not dissociated by extensive dialysis or gel filtration but can be exchanged for Mg-ADP or Mg-ATP in the medium after removal of subunit-ε. A third catalytic site was also known to contain very tightly bound Mg-ATP.

Although these studies preceded the first F_1_ crystal structure, the distances between these nucleotide binding sites and between specifically labeled cysteines on *So*CF_1_ had been determined by FRET measurements (Richter et al., 1985). Because *So*CF_1_ ATPase activity is latent until a disulfide bond on subunit-γ is reduced, each catalytic site could be specifically filled with a VO^2+^nucleotide, and then catalysis-dependent changes could be followed upon activation with dithiothreitol. Rates of VO^2+^-dependent and Mg^2+^-dependent chloroplast F_1_F_O_ ATP synthesis were comparable, and the VO^2+^-dependent F_1_-ATPase activity was higher than that observed with either Mg^2+^, Mn^2+^, or Ca^2+^ (Houseman et al., 1994a).

**Figure 3:**
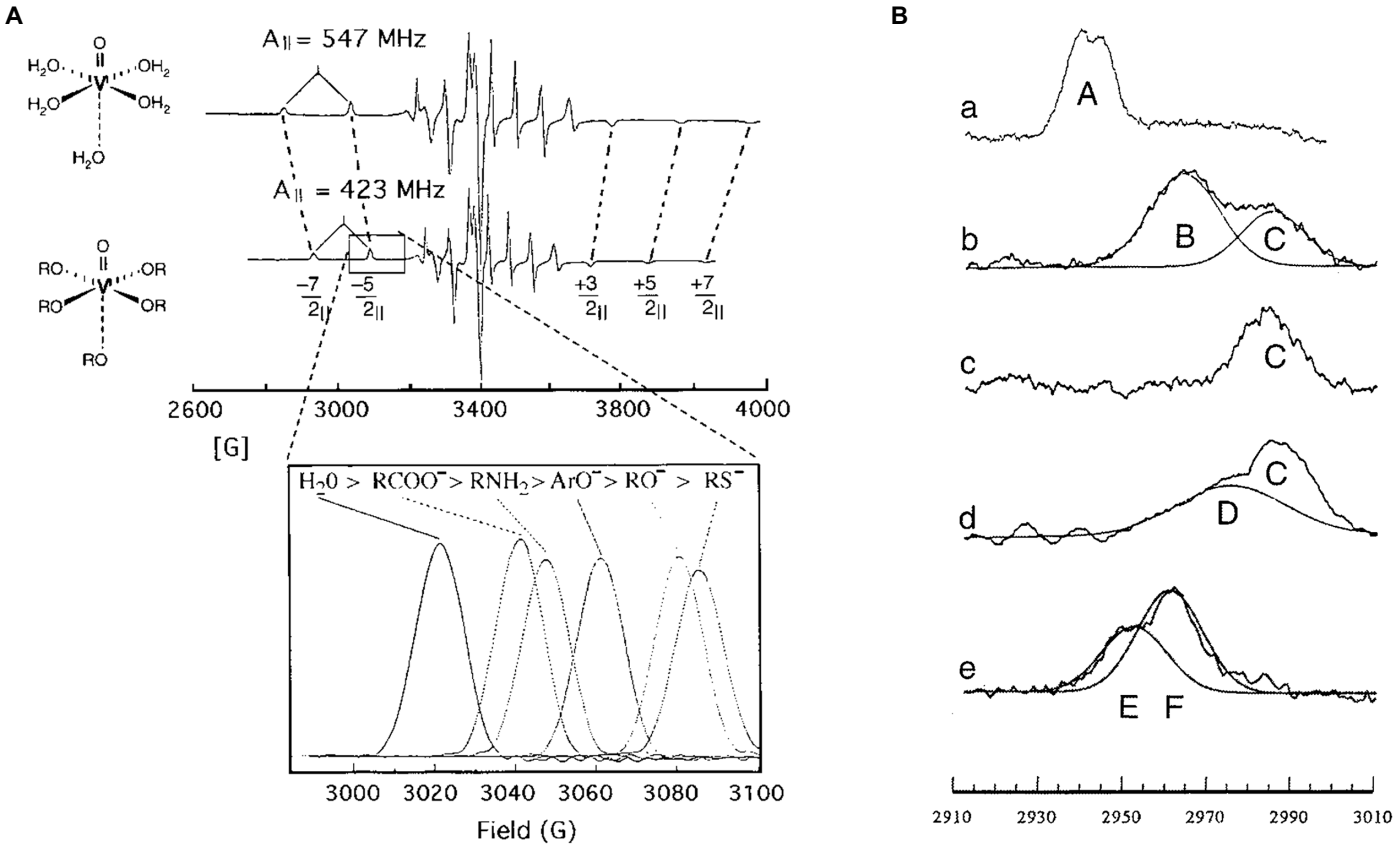
Sequential conformational changes of vanadyl-nucleotides bound to specific F_1_-ATPase catalytic sites demonstrate the F_1_ binding-change mechanism. **(A)** Equatorial ligands determine ^51^V-hyperfine Parameters of VO^2+^. EPR spectra of VO^2+^ with water (top) or hydroxyl groups (bottom) as equatorial ligands. The parallel transitions (−7/2_||_, −5/2_||_, +3/2_||_, +5/2_||_, and + 7/2_||_) that are not superimposed with perpendicular transitions are identified. Inset: dependence of the −5/2_||_ transition on the type of equatorial ligands. **(B)** The −5/2_||_ transition(s) of VO^2+^ bound to *So*F_1_ as: (a) VO^2+^–ATP at latent Site-2, (b) VO^2+^–ADP bound to latent Site-3, (c) VO^2+^–ADP bound to activated Site-3, (d) VO^2+^–ATP bound to activated site 3; and (e) VO^2+^–ATP bound to Site 1 using *So*F_1_–ε. This figure was modified from Frasch (2000a,b) with permission of the publisher.

Vanadyl is composed of the metal V(IV) double-bonded to oxygen that results in a molecule with a net charge of 2+ (Figure 3). Like Mg^2+^, the ligands of VO^2+^ adopt an octahedral configuration with four equatorial ligands and one axial ligand that is *trans* to the oxo group. Vanadyl serves as a sensitive probe of the nucleotide-binding environment of the catalytic sites because each functional group (e.g., phosphate, carboxyl, hydroxyl) coordinated at the equatorial positions contributes independently and quantitatively to the magnitude of the values that define the Electron Paramagnetic Resonance (EPR) spectrum.

The binding of VO^2+^-ATP to non-catalytic Site-2 gave rise to an EPR spectrum designated Species-A (Frasch et al., 1992; Houseman et al., 1994a, 1995a). Filling the low affinity catalytic Site-3 of latent *So*CF_1_ with VO^2+^-ADP or VO^2+^-ATP resulted in an EPR spectrum containing two EPR species, designated B and C, where the former predominated. *So*CF_1_-ATPase is susceptible to inhibition when the metal cofactor binds in the absence of nucleotide (Frasch and Sharp, 1985; Haddy et al., 1985, 1987, 1989). Species B was also observed when VO^2+^ was bound to latent *So*CF_1_ in the absence of nucleotide indicating that this was the cofactor-inhibited conformation in the latent state. Activation of *So*CF_1_ containing VO^2+^-ADP bound to Site-3 resulted in the conversion of species-B to species-C, indicating that the metal ligands had changed (Houseman et al., 1994b, 1995a,b). When VO^2+^-ATP was bound to Site-3, activation not only caused the elimination of Species B, but also formed species-D in addition to species-C, where species-D was the predominant conformation. Species-D represents the environment of VO^2+^-ATP in the tight catalytic site conformation. Thus, consistent with the Binding-Change Mechanism, the binding of VO^2+^-ATP to the low affinity site enabled a large fraction of the enzyme molecules in the sample to change its conformation to the high affinity conformation.

Exchanging VO^2+^-ADP for the Mg-ADP bound to medium affinity catalytic Site-1 resulted in the formation of EPR species-E and -F, where the EPR values of the latter were close to that of VO^2+^ bound in the absence of nucleotide (Chen et al., 2000). Because the Mg-ADP from this site can only be exchanged after activation of F_1_ by removal of subunit-ε, the environment that gives rise to species-E represents the third conformation of the catalytic site. These experiments demonstrated that, at any one time, the metal-nucleotide complex bound to the three catalytic sites are in different conformations, and that the binding of Mg-ATP to the empty site induces the conformations of all three sites to change to that of its successor. Consistent with the Binding-Change mechanism, the concerted conformational changes of EPR species in the three catalytic sites is C → D, D → E, E → F, where in F, nucleotide had dissociated (Frasch, 2000a,b). Based on their binding affinities and positions measured by FRET, these EPR species are analogous to ATPase-driven conformational changes now known as β_E_ → β_T_, β_T_ → β_D_, and β_D_ → β_E_.

Prior to the identification of Mg^2+^ ligands *via* protein crystallography, metal cofactor ligands at chloroplast F_1_ catalytic sites were identified by effects on the EPR spectra of bound VO^2+^-nucleotides from site-directed mutations of *Chlamydomonas reinhardtii* (Hu et al., 1995, 1996, 1999; Chen et al., 1999; Chen and Frasch, 2001; Crampton et al., 2001). Although structures of the *Clamydomonas* chloroplast F_1_ complex are not yet available, recent structures of *So*CF_1_ are consistent with the composition of the amino acid sidechains and phosphate oxygens that comprise coordination environment of the metal cofactor obtained from VO^2+^ EPR spectroscopy.

#### Structural evidence correlating catalytic site occupancy and subunit-γ asymmetry

**Figure 4:**
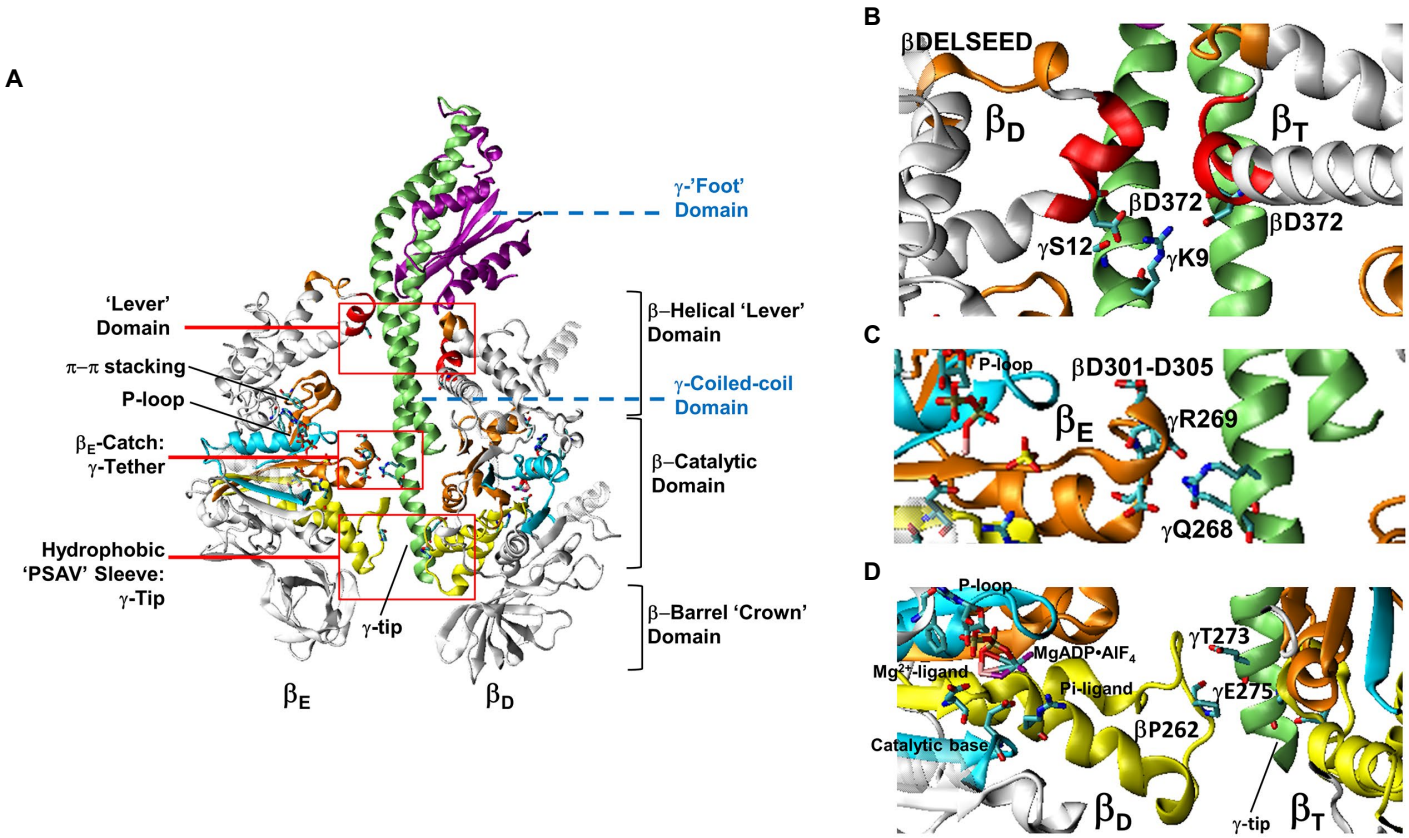
Domain composition of F_1_ showing the relationship between Mg-nucleotide binding motifs and the stator-rotor interfaces. **(A)**. Cross-section of F_1_ showing the relationship between the β_E_ and β_D_-subunit conformations and subunit-γ. Red boxes are detailed in **(B–D)** showing subunit-γ and catalytic site interfaces. **(B)**. Interactions between the β_D_ and β_T_-subunit Lever domains and subunit-γ. **(C)**. Electrostatic interactions between the β_E_-Catch loop and γ-subunit tether residues. **(D)**. The short β_D_ and β_T_-subunit helices that connect the Mg^2+^ and Pi ligands of bound MgADP•AlF_n_ transition state analogs with the hydrophobic sleeve surrounding the C-terminal tip of subunit-γ. Images show *Bt*F_1_ structure pdb-ID 1H8E labeled with equivalent *Ec*F_1_ residue numbers to show the location of bound MgADP•AlF_n_.

The 1BMF (PDB-IDs are used here throughout) structure of *Bos taurus* F_1_ (*Bt*F_1_) provided the first details of the asymmetric relationship of subunit-γ to the surrounding (αβ)_3_-ring, which resulted in the three conformations of the catalytic sites (Abrahams et al., 1994). The α and β subunits, which have closely similar folds, alternate in the ring such that each αβ-heterodimer comprises a catalytic site (Figure 4). Each of these subunits contains a β-barrel “crown” domain, a helical “lever” domain, and a nucleotide-binding domain where, in the β-subunits, ATP is synthesized or hydrolyzed. The crown domains abut to stabilize the catalytic site conformations. The adenine rings of the nucleotides bind to the lever domain *via* π–π stacking with aromatic residues while the nucleotide phosphates bind to the catalytic domain *via* the P-loop such that the γ-phosphate of ATP faces the carboxyl residue that serves as the catalytic base.

Due to the asymmetric interactions of subunit-γ with the ring, the three catalytic sites adopt different conformations (Abrahams et al., 1994). One catalytic site contains bound Mg-ATP (β_T_), the second site contains Mg-ADP (β_D_), while the third site is empty (β_E_). Each of the three non-catalytic sites contains bound Mg-ATP. These conformations were consistent with the VO^2+^-nucleotide studies (Frasch, 2000a,b). The most obvious difference between the conformations of the three β-subunits is that, in the absence of Mg-nucleotide (β_E_), the lever domain is open and extends away from the catalytic domain, whereas it is closed in the β_T_ and β_D_ conformations.

Subunit–γ consists of: (i) a globular “foot” domain that protrudes from the (αβ)_3_-ring and docks with subunit-ε and the c-ring of F_O_; (ii) an antiparallel coiled-coil domain that extends through the core of the (αβ)_3_-ring; and (iii) a singular α–helix at the C-terminal end known as the tip. The (αβ)_3_-ring contacts subunit-γ at three locations. *First*, the ends of the six α– and β–lever domains, which in *Ec*F_1_ β–subunits contain the D_372_IIA sequence, surround and contact the γ-coiled-coil proximal to the globular foot domain. The globular domain of subunit-γ, extends over the closed levers of β_T_ and β_D_ and away from that of β_E_. *Second*, at the end of the γ-coiled-coil distal from the foot, conserved γ–tether residues (*Ec*F_1_-γR268 and γQ269) form strong electrostatic interactions with conserved β_E_–“catch loop” residues (*Ec*F_1_-βD301, βD302, βT304 and βD305). *Third*, the γC-terminal tip residues pass through a hydrophobic sleeve formed by loops of the six α– and β–catalytic domains.

#### Evidence demonstrating F_1_ ATP hydrolysis-dependent subunit-γ Rotation

Although the conformations of the three catalytic sites were staggered, and changed conformations in a concerted, sequential manner, this may have occurred without subunit-γ rotation. Evidence supporting subunit-γ rotation with *Ec*F_1_ was first obtained by mutation βD380C, a point where only one β-subunit could form a disulfide with subunit-γC87 (Duncan et al., 1995). Dissociation of the disulfide crosslinked subunits and reconstitution with ^35^S-labeled β-subunits was followed by disulfide reduction. After ATP hydrolysis, and subsequent crosslinking, the radioactivity of the crosslinked product increased indicating that ATPase activity had randomized the position of the unlabeled β-subunit relative to subunit-γ, consistent with ATP hydrolysis-driven rotation of subunit-γ within the (αβ)_3_-ring. Similar experiments demonstrated rotation with *Ec*F_1_F_O_ (Zhou et al., 1996).

Rotational motion of subunit-γ by at least 200°, as the result of ATP hydrolysis, was also observed in *So*CF_1_ using the technique of polarized absorption relaxation after photobleaching by modifying γC322 with eosin maleimide and immobilizing the *So*CF_1_ on DEAD-A50 Sephadex (Sabbert et al., 1996). The rotational orientations of the subunit-γ bound eosin in the immobilized *So*CF_1_ were evenly distributed prior to a 5 ns polarized light flash, which photobleached the subset of eosin aligned with the direction of polarization. Photobleaching changed the extent of light absorption by eosin (that was probed with continuous 520 nm polarized light) *vs.* time after the flash. This initially reported the fraction of eosin molecules that were photobleached by the laser pulse, but diminished *vs.* time due to *So*CF_1_ ATPase-dependent subunit-γ rotation.

Single-molecule studies of *Geobacillus stearothermophilus* F_1_ (*Gs*F_1_), previously known as thermophilic *Bacillus* Sp. *PS3* F_1_, demonstrated that ATP hydrolysis induced 360° rotation of subunit-γ (Noji et al., 1997). Here, F_1_ was immobilized on a Ni-NTA-coated cover slip by six his-tags on the β–subunit N-terminus that positioned the subunit-γ globular domain distal from the cover slip. Biotinylation of γS107C this globular domain enabled attachment of a 1 μm to 3 μm fluorescently labeled actin filament *via* streptavidin (Müller et al., 2002). As viewed from the foot of subunit-γ, ATPase-dependent counterclockwise (CCW) rotation was observed in discrete 120° steps with a CCD camera at 30 frames sec^−1^. Single-molecule FRET experiments of EcF_1_F_O_ in membrane vesicles confirmed CCW rotation during ATP hydrolysis, and demonstrated clockwise (CW) rotation during ATP synthesis (Diez et al., 2004).

When ATPase-dependent rotation was monitored using a 40 nm gold bead as a visible probe and rotation data of *Gs*F_1_ was collected at 8000 fps, 120° rotational stepping was observed at saturating ATP, which were separated by 2 ms dwells with a kinetic profile that indicated the presence of two successive 1 ms steps (Yasuda et al., 2001). This was designated the catalytic dwell because the duration of the first kinetic step of the dwell was extended by ATPγS (adenosine 5′-[γ-thio]triphosphate) or by mutation of catalytic base residue βE190D, which each slow ATP hydrolysis. The increase in duration of the second kinetic step with Pi indicated that Pi release ends the catalytic dwell and rotation resumes. At ATP concentrations that limited the rate of ATPase activity, a second ATP-binding dwell was observed 30°–40° after the catalytic dwell. The duration of this dwell varied inversely with ATP concentration.

Quantitation of tryptophan fluorescence quenching as a function of ATP binding to *Ec*F_1_ established that the affinity of the three catalytic sites differed by several orders of magnitude (Weber et al., 1993) consistent with the nucleotide affinities of *So*CF_1_ (Bruist and Hammes, 1981). Subsequent *Ec*F_1_ tryptophan fluorescence studies using ITP, which binds with lower affinity than ATP, showed that two catalytic sites always contain bound nucleotide, and that ATP binding to the third site induces catalysis (Weber and Senior, 1997, 2001). This 3-site mechanism was confirmed in single-molecule rotation studies using fluorescent Cy3-ATP, which showed that the ATP that bound to a catalytic site at 0° remained bound for 240°–320° during forced rotation of a magnetic bead probe by an external magnet (Adachi et al., 2007). However, these experiments were unable to resolve the precise rotational position of ADP release.

#### Use of gold nanorods to measure F_1_ ATPase-driven rotational power strokes

The single-molecule rotation studies with actin filaments and gold beads provided important information regarding the rotational positions of the catalytic dwell and the ATP-binding dwell. However, as the result of limitations on the frame rate at which rotational data could be acquired, and precision of the rotational position during an ATPase-driven power stroke, information was scarce concerning the periods between the dwells when subunit-γ was in the process of rotating. As a result, the rotation appeared to occur as a discontinuous function between dwell positions.

**Figure 5:**
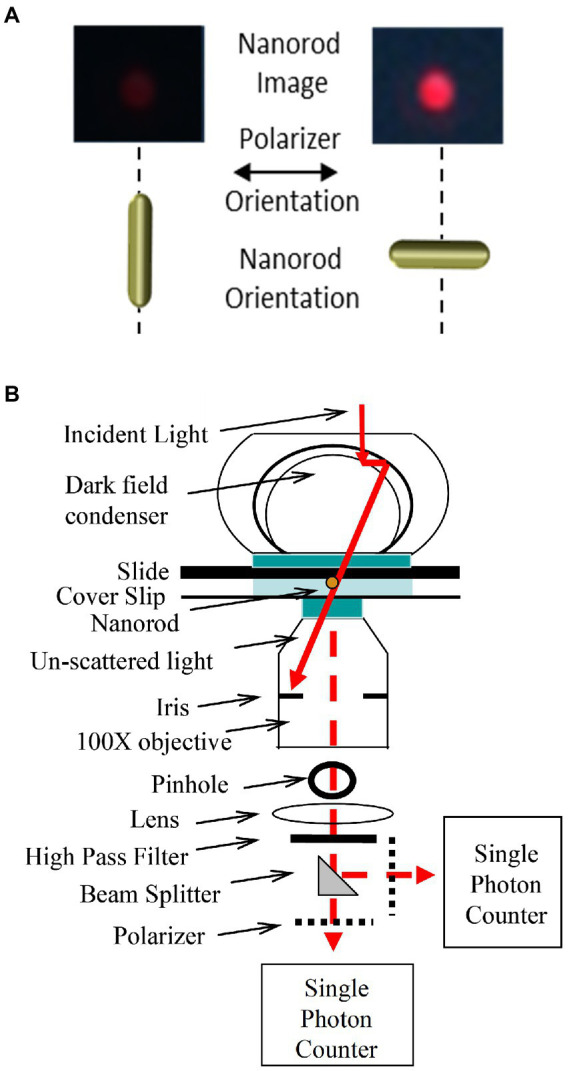
Use of scattered red light intensity from a 75 × 35 nm gold nanorod (AuNR) to measure rotation at the nanoscale. **(A)**. Color image of AuNR-scattered light viewed by dark-field microscopy through a high pass filter to eliminate all but red light and a polarizer filter when the long axis of the AuNR is perpendicular and parallel to the plane of polarization. **(B)**. Dark-field microscope design to record changes of scattered red light intensity vs. time from a single AuNR capable of determining the direction of rotation using a beam splitter to divert half of the photons through a second polarizer set at a rotational position that will reach a maximum light intensity before the first if the AuNR is rotating counterclockwise (CCW). This figure has been modified from that in Hornung et al. (2011).

To capture subunit-γ rotation between dwells, the intensity of polarized light scattered from a gold nanorod (AuNR) was measured by a single-photon counting avalanche photodiode (Spetzler et al., 2006; Hornung et al., 2011). Identification of AuNR attached to rotating F_1_ in the presence of 1 mM MgATP were initially identified in a field of view of the microscope through a polarizing lens with a color camera at 50 fps (Figure 5). At this data acquisition rate, rotating AuNR blinked red and green as the long and short axes, respectively, became aligned with the polarizer. In contrast, the color did not change for any AuNR that was not rotating. Single-molecule F_1_-ATPase-dependent AuNR rotation was then interrogated by aligning it with a 100 μm pinhole that eliminated all light except that which was scattered from the selected AuNR (Hornung et al., 2011). The scattered light was then passed through a polarizing filter mounted on a rotational stage, a high wavelength pass filter to eliminate all but red light that was focused onto a single-photon counting avalanche photodiode (Perkin-Elmer SPCM-AQR-15). The detector had a dark count of ~50 photons/s with a temporal resolution of 50 ns.

**Figure 6:**
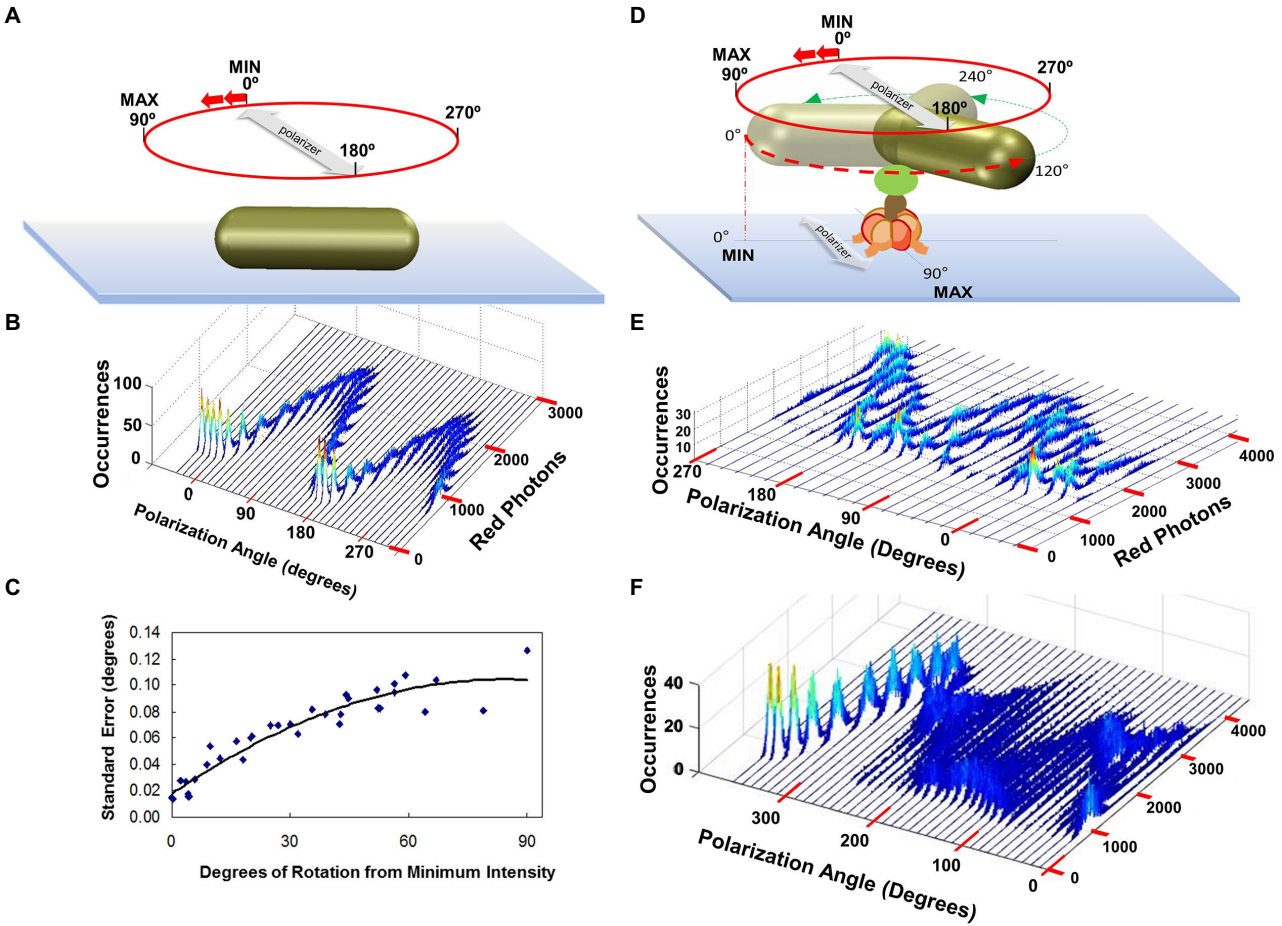
Polarizer Rotation Measurements (PRM) to determine rotational position measurement error and to show F_1_-ATPase-dependent 120° step rotations. **(A)** PRM measurement of a nonrotating AuNR attached directly to the cover slip. Red photons scattered from the AuNR were measured in 5 s intervals at a data sampling rate of 1 kHz (1,000 fps) at each 10° stepped rotational position of the polarizer. **(B)** PRM-dependent distribution of photons scattered from the non-rotating AuNR of **A** vs. rotary position of the polarizer. **(C)** Standard error of the AuNR rotary position vs. degrees of rotation from the minimum intensity calculated from **B**. This error is equivalent to that obtained from a data set of 3,250 power strokes. **(D)** PRM measurement of a AuNR attached to an actively rotating subunit-γ of the F_1_-ATPase in the presence of saturating ATP concentrations. **(E)** PRM-dependent distribution of photons scattered from the non-rotating AuNR of **A** vs. rotary position of the polarizer. **(F)** PRM when the AuNR is attached to actively rotating F_1_ subunit-γ, which stops rotating at polarizer angle 260°, likely due to subunit-ε inhibition. This figure was reconfigured from Spetzler et al. (2006), Ishmukhametov et al. (2010), Sielaff et al. (2016).

The intensity of light scattered from a AuNR changes in a sinusoidal manner as a function of the rotary position of the AuNR relative to the plane of polarization with minimal and maximal intensities separated by 90° (Spetzler et al., 2006). The distribution of scattered red light intensities from a single AuNR immobilized to the surface of a microscope slide as a function of the rotational position of the polarizing filter is shown in Figures 6A,B (Ishmukhametov et al., 2010). At each rotary position of the polarizer, the scattered light intensity is sampled 3,520 times under conditions comparable to that used to measure rotation of single F_1_ ATPase molecular motors. This sample number was used because it corresponds to the average number of F_1_-ATPase power stroke events during a 5 s or 50 s data acquisition period for a given F_1_ molecule or F_1_F_O_ molecule rotation measurement, respectively, when data is collected at 100 kHz (equivalent to 100,000 fps). The scattered light intensity from the AuNR varied between maximum and minimum values of 2,500 and 500 photons. The difference between these values comprises a dynamic range of ~2000 photons per sample, which determines the sensitivity of the measurement. This was the minimum dynamic range used to measure rotation, while the average range was ~3,000 per sample. Thus, calculation of the error from these data represents an upper limit in a rotation data set acquired from rotation of a single F_1_ molecule. The distribution of light intensities scattered from the AuNR was smaller at polarization angles in which the intensity was at a minimum than the distribution at maximum intensities. The standard error in the measurements at each rotary position of the polarizer varied between 0.02° and 0.12° degrees as calculated from the minimum and maximum intensity values (Figure 6C).

*Polarizer Rotation Measurements show 120° step rotations at saturating Mg-ATP.* In a polarizer rotation measurement (PRM), the variation of intensity of red light scattered from a nanorod attached to a single F_1_ molecule in the presence of saturating MgATP was observed as a function of the stepped rotation of the polarizer by 10°, in 5 s intervals, at a data sampling rate of 1 kHz (Spetzler et al., 2006). This low data acquisition speed reports the positions of the three catalytic dwells because subunit-γ rotation during the power stroke typically occurs too fast (μsec time scale) for the detector to capture most of the intermediate rotational AuNR positions (Figures 6D,E). Each dwell contributes a peak in the distribution of the histogram at a given set angle of the polarizer. When viewed as a series of histograms of light intensities at each of 36 polarizer angles covering 360°, three offset sinusoidal curves in scattered light intensities were observed from a nanorod attached to the actively rotating drive shaft of a single F_1_-ATPase. Because the dependence of light intensity versus AuNR orientation relative to the axis of the polarizer is sinusoidal, the three offset sinusoidal curves in the histogram indicates rotation occurs in 120° power strokes separated by catalytic dwells.

For a given F_1_, the spacing between the three sinusoidal curves in a PRM is sensitive to the tilt of the rotation axis of subunit-γ from orthonormal to the microscope cover slip. Given that the data acquisition speed is set at 1 kHz, the variation in intensity of catalytic dwells *vs.* the rotary position of the polarizer indicates that catalytic dwells are shorter at some positions. When light intensity is observed in the space between the three sinusoidal curves, the angular velocity of the power stroke is slow enough to be captured by the 1 kHz data acquisition speed. To date, the three off-set sinusoidal intensity curves observed by PRMs, which are indicative of three successive 120° power strokes, have been observed with *Ec*F_1_ (Spetzler et al., 2006; Sielaff et al., 2016), *Gs*F_1_ (Sielaff et al., 2016), and *Mycobacterium smegmatis* F_1_ (*Ms*F_1_) (Ragunathan et al., 2017), as well as with the *Methanocarcina mazei Gō1* A_1_-ATPase (*Mm*A_1_) (Sielaff et al., 2016).

*Rotation temporarily stopped by ε-subunit or Mg-ADP inhibition.* Rotation catalyzed by *Ec*F_1_ is subject to intermittent inhibition by the C-terminal helical domain of subunit-ε (εCTH) or by Mg-ADP, each of which can last for seconds (Sekiya et al., 2010; Shah et al., 2013). These inhibitions are independent processes, where the former results from the extension of the εCTH along the coiled-coil during the catalytic dwell that competes with the possible transition into the Mg-ADP inhibited state (Shah et al., 2013). In all single-molecule AuNR rotation assays, substrate was added as a 2:1 ratio of ATP:Mg^2+^, which is known to minimize the occurrence of Mg-ADP inhibition (Hyndman et al., 1994; Kato et al., 1995). About 25 and 50% of the molecules in a given field of view were observed to rotate in the presence of MgATP and MgGTP, respectively (York et al., 2007). This difference is explained by the fact that hydrolysis of GTP is less sensitive to this type of inhibition (Hyndman et al., 1994). In comparison, ~5% of the F_1_-ATPase molecules are observed to rotate using a gold bead assay, which is likely because observation of rotation requires precession of the bead around the axis of rotation (Yasuda et al., 1998).

During a PRM assay, inhibition by subunit-ε or Mg-ADP is evident by the temporary conversion of the three off-set sinusoidal intensity curves to a single intensity curve (Figure 6F) that lasts until inhibition is relieved and rotation resumes. Inhibition by Mg-ADP occurs at catalytic dwell positions (Hirono-Hara et al., 2001; Sekiya et al., 2010; Bilyard et al., 2013), and pauses can last ~30 s in *Gs*F_1_ (Hirono-Hara et al., 2001). Using the AuNR assay, the average catalytic dwell duration with saturating 1 mM MgATP was 8.3 ms for an *Ec*F_1_ preparation with a k_cat_ of 130 s^−1^, which was measured by an ensemble ATPase coupled assay (Spetzler et al., 2006). This k_cat_ value corresponds to 7.7 ms per ATP hydrolyzed, which was comparable to the average dwell duration for all dwells including the long dwells that result from ε-subunit or Mg-ADP inhibition. The duration of *Ec*F_1_ catalytic dwells averaged ~2 ms during periods that where extended inhibition dwells were absent (Nakanishi-Matsui et al., 2006), which are comparable to those observed with *Gs*F_1_ (Shimabukuro et al., 2003).

*Resolution of F_1_ rotary power stroke positions between catalytic dwells.* The charge-coupled device used to quantify the number of photons scattered from a single AuNR has a 50 nsec time resolution. Consequently, to obtain light intensity measurements at 1 kHz, the number of photons measured were binned in successive 1 ms intervals. Due to the brightness of the AuNR with the light source used, it was possible to resolve F_1_ATPase-driven rotational position *vs.* time when scattered light intensity was sampled at rates as high as 400 kHz, corresponding to 2.5 μs per data point (Spetzler et al., 2006).

**Figure 7:**
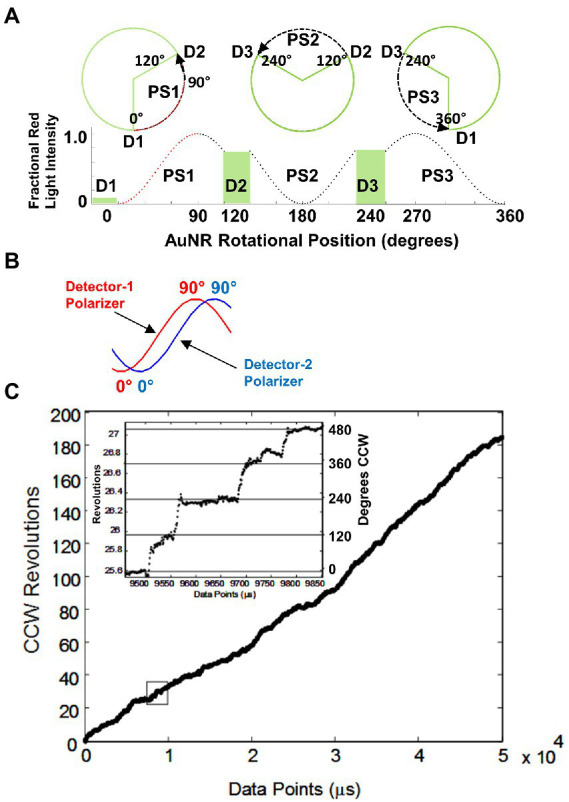
**(A)** Changes in AuNR scattered red light intensity during one complete revolution involving three consecutive power strokes (PS) and three consecutive catalytic dwells (D) separated by exactly 120° when, prior to data collection, the polarizer is rotated such that scattered light intensity is at a minimum during catalytic dwell-1 (D1). **(B)** Rotational off-set of polarizer positions when two detectors are used to observe the direction of rotation. **(C)** Counterclockwise rotation observed by *Ec*F_1_-ATPase-dependent rotation calculated from two detectors with off-set polarizer positions with data acquired at 10 kHz in the presence of 1 mM MgCl_2_ and 2 mM ATP. (Inset) Detail of the rotational stepping of the boxed region. Horizontal lines show the 120° dwell positions. This figure was reconfigured from Spetzler et al. (2009).

These measurements were the first to reveal details of the position of the F_1_ axle as it rotated between catalytic dwells (Martin et al., 2014). To accomplish this, the changes in intensity of AuNR scattered light from a rotating F_1_ molecule measured by the avalanche photodiode is examined for the minimum and maximum scattered light intensity difference *vs.* time (Hornung et al., 2011). The polarizer is then rotated to maximize this difference, which aligns the polarizer with the short axis of the AuNR, such that the light intensity is at a minimum during one of the three catalytic dwells. As a result, the light intensity increases from a minimum during the subsequent power stroke, which passes through a maximum intensity upon rotation by 90°, then decreases in intensity until the next catalytic dwell begins at 120° (Figure 7A). A power stroke is defined here by the rotation of subunit-γ between catalytic dwells. During the second power stroke, the intensity passes through the minimum but not through the maximum, and during the third, the intensity first passes through the maximum upon rotating 30° and returns to the minimum intensity as a 360° rotation is completed.

After collecting a data set for 5 s from each F_1_ molecule at 200 kHz, rotation events that began at a minimum and passed through a maximum were collected for further analysis (Spetzler et al., 2006). Data sets were analyzed that each comprised ~3000 of these 120 degree power strokes, which undergo F_1_ ATPase-dependent rotation in the absence of subunit-epsilon and/or Mg-ADP inhibition.

*EcF_1_-ATPase-dependent rotation is CCW.* To determine whether *Ec*F_1_ rotated exclusively CCW when actively hydrolyzing ATP, a beam splitter was placed in the path of scattered the red light scattered from a rotating AuNR and each beam was directed through a separate polarizer and photon counter for quantification (Spetzler et al., 2009). The polarizers were set a different rotary positions such that the direction of AuNR rotation was determined by the photon counter that measured the maximum intensity of scattered light first (Figure 7B). All *Ec*F_1_ molecules examined rotated almost exclusively CCW for the length of the measurements, during which subunit-γ completed ~200 revolutions equivalent to a total of 600 consecutive power strokes (Figure 7C). Similar CCW rotation has been observed with *Gs*F_1_ during ATP hydrolysis (Noji et al., 1997).

*Average torque generated by EcF_1_ during a power stroke.* Torque is a measure of the ability of a motor to rotate against an opposing load. To measure the average torque during a power stroke, the load on *Ec*F_1_ was increased by varying the viscous drag on the AuNR attached to subunit-γ (Hornung et al., 2008). The effects of drag *vs.* the time required to rotate between catalytic dwells, and the duration of these dwells in the presence of saturating (1 mM) Mg-ATP was then determined (Spetzler et al., 2009). To vary viscous drag, rotation was compared when subunit-γ was attached to a AuNR with dimensions of 73 × 35 nm, 87 × 36 nm, 90 × 46 nm, and 91 × 45 nm. Although the latter two were of similar dimensions, the former had a rectangular profile, while the latter had rounded ends. The effects of load on *Ec*F_1_ rotation were also measured when viscous drag was varied by the addition of polyethylene glycol 400 (PEG-400). The viscosity of the buffers containing PEG-400 (vol/vol) were measured directly, and the data were used to calculate the shear stress *vs.* shear rate. The linear dependence between these parameters indicated that the assay buffer containing PEG-400 behaves as a Newtonian fluid. As a result, the PEG-400 molecules are too small to be pulled along by the rotating AuNR, and do not make secondary nonlinear contributions to the drag.

**Figure 8:**
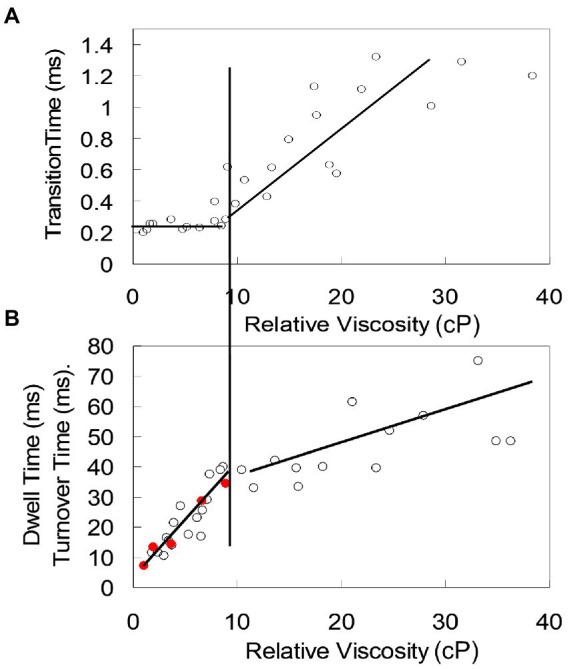
Interdependence of catalytic dwell duration, ATPase activity, and average transition time (time required for the power stroke to rotate 90°) as a function of load on the *Ec*F_1_ motor. **(A)** Transition time vs. viscosity. **(B)** Catalytic dwell duration (○) and ensemble ATPase activity measured as k_cat_ (●) vs. viscosity. Load on the motor was varied by increasing concentration of PEG-400, which increased the viscous drag on the AuNR attached to subunit-γ. At loads <4 aN nm ms (vertical line between **A** and **B**), transition time is constant (not load-limited) while catalytic dwell time and k_cat_ show the same proportional increase with drag. At loads >4 aN nm, both transition time and catalytic dwell time increase proportionately with load. These data were reconfigured from those in Spetzler et al. (2009).

When any of the three smallest AuNR were bound to subunit-γ, the average time for the power stroke to rotate 90° from the end of the catalytic dwells (transition time) was constant (~275 μs) until the load on the motor reached 4 aN nm ms (Spetzler et al., 2009). This indicated that the velocity of the rotation between catalytic dwells was not limited by viscous drag on the AuNR under these conditions, but instead by the intrinsic rate-limiting properties of the *Ec*F_1_ motor. At higher viscosities, the transition time increased proportional to the drag (Figure 8). However, the duration of the catalytic dwell and the ensemble measurement the turnover time (k_cat_) of ATPase-activity increased proportionately with drag on the motor at loads <4 aN nm ms. At higher loads the proportional increase in catalytic dwell time changed concurrent with the dependence of average transition time *vs.* drag. This strongly suggests that the rate of ATP hydrolysis and/or Pi release, which occur during the catalytic dwell (Spetzler et al., 2009), involves motion of subunit-γ that is dampened by the imposed load.

Torque was calculated using the 91 × 45 nm nanorods that imposed significant drag on the rotation in the absence of PEG-400 (Hornung et al., 2008). The drag coefficient was determined directly by measuring the amount of angular change of individual AuNR that were suspended near, but not attached to the surface of a microscope slide in the absence of *Ec*F_1_, which enabled calculation of the diffusion coefficient. Using Einstein’s relation, the diffusion coefficient was used to determine the drag coefficient.

The average torque generated by *Ec*F_1_ varied as a function of viscous load on the AuNR, which averaged 63 ± 8 pN nm (Hornung et al., 2008). A statistically equivalent torque value of 56 ± 6 pN nm was obtained with *Ec*F_1_ (Junge et al., 2009), and of 50 ± 6 pN nm with *Ec*F_1_F_O_ (Pänke et al., 2001), using the extent of deformation of an actin filament attached to the rotor. Torque values of ~80 pN nm and 40–50 pN nm for *Gs*F_1_ were estimated from actin filament (Yasuda et al., 1998) and duplex bead rotation rates (Usukura et al., 2012).

*Rotor-Stator contact points contribute to F_1_-ATPase activity and rotation.* The mechanism in which the energy generated by the consumption of ATP is used to power subunit-γ rotation, and conversely, the means that by which rotation powered by F_O_ acts upon the catalytic sites in the (αβ)_3_-ring to synthesize ATP continues to be the focus of intense investigation. The interactions between the (αβ)_3_-ring and subunit-γ responsible for F_1_-ATPase-powered rotation have alternatively emphasized electrostatic interactions (Mukherjee and Warshel, 2011, 2015), steric interactions (Koga and Takada, 2006; Pu and Karplus, 2008), or an elastic spring mechanism between the stator and rotor (Czub and Grubmüller, 2011). The results described here indicate that all these interactions contribute to the rotation mechanism.

Mutations that alter interactions of charged and polar groups between subunit-γ and the (αβ)_3_-ring were examined to assess their contributions to catalytic activity and ATPase-dependent rotation (Greene and Frasch, 2003; Boltz and Frasch, 2005, 2006; Lowry and Frasch, 2005). These included the (αβ)_3_-hydrophobic sleeve that surrounds the tip comprised of the C-terminal helical extension beyond the coiled-coil, the β_E_-catch loop electrostatic interactions with the γ-tether residues on the C-terminal helix of the coiled-coil distal from the foot, and the (αβ)_3_-lever domains that surround the γ-subunit coil-coil proximal to the globular foot domain.

***The hydrophobic sleeve.*** The subunit-γ C-terminal tip consists of a single α–helix that extends 17–19 amino acids (depending on species) through the (αβ)_3_-hydrophobic sleeve. The β-subunit loops of the sleeve contain the conserved PSAV motif, where βP262 in *Ec*F_1_ constrains the mobility of the loop (Figure 4D). The β_D_ and β_T_ loops contact the final 12 and 13 residues of the tip, respectively, while the β_E_-loop contacts subunit-γ 9 and 15 residues from the C-terminus. Residues γE275 and γT273 are the only polar groups in the γC-terminal tip that pass through the hydrophobic sleeve.

Single-site *Ec*F_1_ mutations of βP262 and βV265 in the PSAV loops, and mutations of γE275 and γT273 in the subunit-γ tip were made to determine their impact on enzymatic function (Boltz and Frasch, 2005). The γT273D and γE275D mutations increased purified *Ec*F_1_ ATPase activity >1.5-fold, increased ATPase-dependent proton gradient formation by *Ec*F_1_F_O_ in inverted membranes measured by ACMA quenching, and increased ATP synthesis measured by growth on succinate. Conversely, mutants βP262G, γT273A, and γT273V decreased *Ec*F_1_ ATPase activity by ~2 orders of magnitude, abolished ATPase-dependent proton gradient formation by *Ec*F_1_F_O_ in inverted membranes, and decreased ATP synthase measured by growth on succinate.

The ATPase activity of *Ec*F_1_ is inactivated by the binding of the transition state analog MgADP•AlF_n_ that forms over a period of ~240 min upon addition of Mg-ADP, AlCl_3_, and NaF (Boltz and Frasch, 2005). Mutations γT273A, γT273V, and βP262G decreased the rate of *Ec*F_1_ ATPase inactivation due to the binding of MgADP•AlF_n_, where the latter mutation increased the rate by 3 orders of magnitude. Conversely, mutations γT273D and γE275D increased the rate of inactivation by MgADP•AlF_n_ consistent with the increase in catalytic activity of the enzyme.

These studies (Boltz and Frasch, 2005) were the first to show that specific interactions between subunits-γ and -β are linked to ATP hydrolysis and ATP synthesis. Catalytic residues βD242 and βR246 in *Ec*F_1_, which serve as a Mg^2+^ ligand and bind Pi upon hydrolysis of ATP, respectively, are connected to the PSAV loop *via* a short α-helix. In the “transition state” *Bt*F_1_ structure 1H8E (Menz et al., 2001), γE275 and γT273 form hydrogen bonds to the backbone of βV265 of catalytic sites β_T_ and β_D_, respectively, that both contain the bound transition state analog MgADP•AlF_4_^−^. Consequently, the mutations examined were found to either increase or decrease the ability to form the transition state that results in ATP hydrolysis.

***The Catch Loop.*** In the β_E_ conformation, a strong electrostatic interaction exists between carboxyl residues in the “catch loop” with residues of the γ-subunit tether at the end of coiled-coil that are separated from the tip residues that pass through the hydrophobic sleeve by several amino acids (Figure 4C). This electrostatic interaction exists in every F_1_ structure solved to date. Consequently, this interaction identifies the β_E_ conformation of the motor, despite differences in its nucleotide occupancy or the open or closed position of the β-lever domain.

Selective mutations of individual *Ec*F_1_ residues that diminished or eliminated electrostatic interactions between the β_E_-conformation catch loop (residues 301–305) and the subunit-γ tether (γR268, γQ269) were also found to dramatically decrease ATPase activity of purified F_1_ (Greene and Frasch, 2003). Of these, βD302V, βD305V, βD305S, abolished F_1_-ATPase activity. The ATPase activity of the conservative βD305E mutation, which retained a carboxyl group, as well as βD302T, and γQ269L decreased by ~2 orders of magnitude, while that of γR268L decreased 10-fold from that of WT.

In the β_T_ and β_D_ conformations, catch loop residues βD302 and βD305 form electrostatic interactions with residues αR283, from the respective α_T_ and α_D_-subunits, while catch loop βD301 residues form electrostatic interactions with β_T_R323 and β_D_R323. Mutation αR282E eliminated ATPase activity while αR282Q and βR323K decreased it by ~10-fold (Boltz and Frasch, 2006).

Mutations that eliminated ATP synthase activity as measured by growth on succinate included γQ269L, βD301E, βD305V, βD305S, and βD305E, while mutants γR268L, βD301V, βD301T, βD301N, βD302V, and βD302T decreased the growth rate several fold (Greene and Frasch, 2003; Boltz and Frasch, 2006). These results clearly showed that these electrostatic interactions are extremely important for ATPase activity, and perhaps are essential for *Ec*F_1_F_O_ to catalyze ATP synthesis. Based on these results, these residues were proposed to act as an escapement mechanism that insures tight coupling of substrate binding to the concerted conformational changes during the alternating site mechanism (Greene and Frasch, 2003), which essential for all motors and clocks. During F_1_ ATPase-driven rotation, ATP binding would trigger release of the β_E_-catch allowing CCW rotation of subunit-γ, which induces the subunit-γ tether to connect with the β_T_ catch such that β_E_ → β_T_, β_T_ → β_D_, and β_D_ → β_E_.

***The β-Levers.*** The (αβ)_3_-lever domains that surround the γ-subunit coil-coil are comprised of a helix-turn-helix (Figure 4B). The *Ec*F_1_ βD_372_IIA sequence motif at the C-end of the first helix of each β-lever contact γ-coiled-coil residues proximal to the γ-globular foot domain. This motif is followed by the DELSEED sequence in the β-lever turn, which does not contact subunit-γ, and has been shown not to contribute significantly to the rotational or catalytic mechanism (Hara et al., 2000).

The effects of mutations of *Ec*F_1_ residues of β-lever residue βD372V, and γ–subunit contacts γK9I, γS12A and double mutation γK9I/S12A were examined for their impact on enzymatic function (Lowry and Frasch, 2005). Mutants βD372V, γS12A and γK9I/S12A reduced ATP synthase activity by 2 orders of magnitude as measured by growth on succinate. Mutations βD372V, γK9I, γS12A and γK9I/S12A all abolished the ability of F_1_F_O_ to catalyze ATPase-dependent proton pumping. In contrast, γK9I and γK9I/S12A decreased ATPase activity 2-fold, while those of γS12A and βD372V was essentially unchanged from that of WT. This shows that, while these mutations do not decrease ATPase activity in the absence of a load, the presence of a pmf (which applies an opposing load) decreases the ATPase rate significantly. In other words, these mutations decrease the ATPase-dependent torque significantly.

The results that demonstrate that the interactions of subunit-γ with both the hydrophobic bearing (Boltz and Frasch, 2005) and the catch loop (Greene and Frasch, 2003; Boltz and Frasch, 2006) contribute significantly to the F_1_-ATPase catalytic mechanism have been confirmed by studies of the effects of subunit-γ truncation mutants on rotation and catalysis (Furuike et al., 2008; Hossain et al., 2008). In these single-molecule studies, truncation of the γC-terminus by as few as 14 residues decreased ATPase activity significantly. Deletion of 17 residues, which made the subunit-γ tip too short to extend through the hydrophobic sleeve, decreased ATPase activity by an order of magnitude. Elimination of the γ-subunit tether by a 21-residue truncation decreased ATPase activity by 2 orders of magnitude and resulted in a 2-fold decrease in torque.

The number of molecules with truncations of ≥35 residues of the γC-terminus with a truncation of the γN-terminus to match the length of the coiled-coil had ATPase activities almost 3 orders of magnitude lower than WT (Furuike et al., 2008; Hossain et al., 2008). The F_1_ molecules that were observed to rotate became exceedingly rare (fewer than 1 per field of view) with movements that primarily stumbled forward and backward rather than rotating. It is noteworthy that an F_1_ molecule can occasionally appear to be catalyzing ATPase-dependent rotation, but the movement is the result of a loose attachment of the His-tags to the surface of the cover slip.

It is likely that the inability of molecules to rotate that contain truncations of ≥35 residues result from deletions of the section of the coiled-coil that interact with the β-lever. The β_T_-DIIA lever motif contacts γC-helix residues γ252 and γ256, and β_D_-DIIA contacts γN-helix residues γK9 and γS12 when *Ec*F_1_ is not in the ε-subunit inhibited state. A 35-residue truncation deletes both γ252 and γ256. The position that the β_E_-DIIA lever contacts the γ-coiled-coil varies among F_1_ structures. Example subunit-γ contacts with the β_E_-DIIA using *Ec*F_1_ numbering include: γN-helix-γ15 and γ17, “transition state” structure 1H8E (Menz et al., 2001); γN-helix-γ25, “ground state” as well as “hydrolysis dwell” structures 2JDI and 7L1R (Bowler et al., 2007; Sobti et al., 2021); γC-helix-γ243 and γ244, structure 4ASU (Rees et al., 2012); γC-helix-γ227-γ228, “ε-inhibited” structure 3OAA (Cingolani and Duncan, 2011); and γC-helix-γ231, “ADP-binding” structure 7L1Q (Sobti et al., 2021).

In addition to showing that both the hydrophobic sleeve and the catch loop interactions were important for catalysis and rotation, these results also confirmed that the catch loop interaction is responsible for one half of the torque generated by F_1_. This suggests that the other half is derived from the β-lever interactions with subunit-γ. The results that demonstrate that the interactions between subunit-γ and the β-lever DIIA motif (Lowry and Frasch, 2005) contribute significantly to the *Ec*F_1_-ATPase catalytic mechanism have been confirmed by studies of the effects of mutants *Gs*F_1_ that truncated portions of the β-lever that included the DI of the DIIA motif on rotation and catalysis (Usukura et al., 2012). These truncations decreased the rate of ATP synthesis by ~10-fold and significantly decreased the ability of F_1_F_O_ to catalyze ATPase-dependent proton pumping in inverted membranes. The truncations also decreased torque generated by the F_1_-ATPase by 2-fold. The ATPase activity of the *Gs*F_1_ motors in the absence of viscous load was not very different from WT. However, in the presence of a load such as rotating large beads and/or pumping protons to create a pmf, the decrease in torque was apparent.

These results closely replicate the effects of *Ec*F_1_ β-lever βD372V and the complimentary mutations of one of its contact sites on subunit-γ (Lowry and Frasch, 2005). Taken together with the results of mutations that demonstrate that contribution of the interactions between the catch loop and the subunit-γ tether, clearly half of the torque is generated by the β-lever interaction and half of the torque is generated by the catch loop interaction, while the hydrophobic sleeve interaction is contributes significantly to the rate of transition state formation for ATP hydrolysis.

Although their data show that the tip and the coiled-coil of subunit-γ make significant contributions to the production of torque, Hossain et al., 2006, 2008 concluded that neither helix in the coiled-coil region of the axle of F_1_-ATPase plays a significant role in torque production. Other more radical chimera of F_1_ have been observed to rotate in some manner (Mnatsakanyan et al., 2009; Kohori et al., 2011; Chiwata et al., 2014). However, more work is required to clarify what insight these chimeric complexes can provide to the mechanism of the F_1_ ATPase and intact F_1_F_O_.

**Figure 9:**
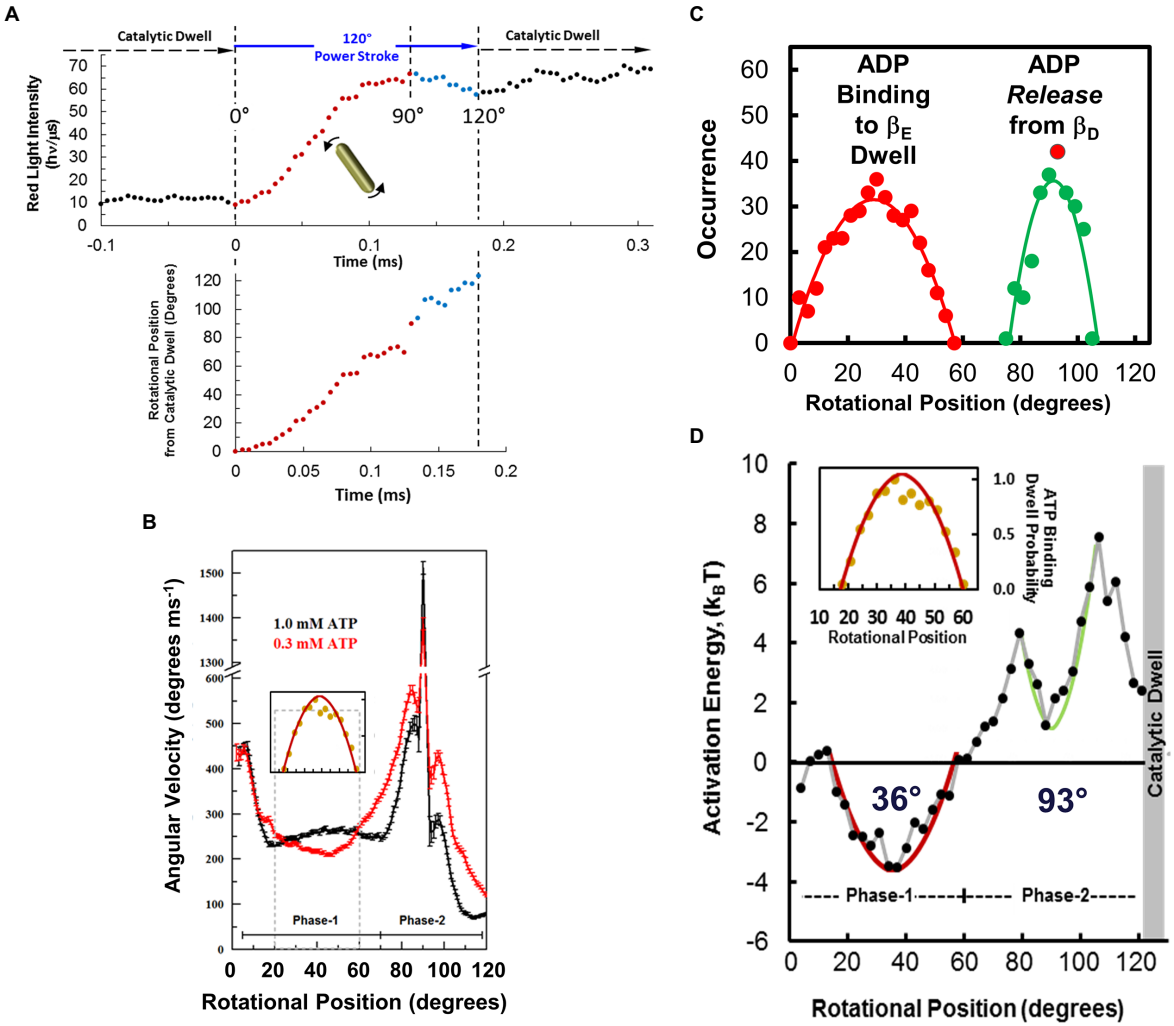
Measuring F_1_-ATPase power stroke angular velocity vs. rotary position from red light intensity of a rotating AuNR collected at 200 kHz (200,000 fps). **(A)**. Example of changes in scattered light intensity vs. time during an *Ec*F_1_ power stroke in saturating 1 mM Mg-ATP. This power stroke occurred subsequent to the catalytic dwell, which had been set to a minimum light intensity by rotating the polarizer prior to data collection. Power stroke rotational position vs. time was calculated from scattered light intensity using Eq. 1 (red) and Eq. 2 (blue). **(B)**. Average power stroke angular velocity vs. rotational position of *Ec*F_1_ ATPase-driven γ-subunit rotation binned for every 3° of rotation from the end of the catalytic dwell, which was designated as 0° in the presence of saturating 1 mM Mg-ATP (black) and 0.3 mM Mg-ATP (red). Inset: Distribution of ATP-binding dwells vs. rotational position in the presence of 0.3 mM Mg-ATP, which is proportional to the decrease in average angular velocity vs. rotational position at 0.3 mM Mg-ATP. **(C)**. Distributions of ADP-binding dwells (red) and ADP-dependent velocity decreases (green) vs. rotational position of the power stroke at 1.0 mM MgATP and 3 mM MgADP. **(D)**. Activation energy of the *Ec*F_1_-ATPase power stroke vs. rotational position at 1 mM Mg-ATP, 10 μM Mg-ADP and 10 μM Pi. Energy stored vs. extent of twist of a torsion spring with spring constants, κ = 50 k_B_T rad^−2^ (red) and κ = 150 k_B_T rad^−2^ (green) from equilibrium positions of 34° and 93°, respectively. Inset: Distribution of ATP-binding dwells vs. rotational position from B. This figure was reconfigured using data from Martin et al. (2014, 2018), Sielaff et al. (2016).

*F_1_ and A_1_ ATPase Power Stroke Angular Velocities* *vs.* *Rotational Position.*Using AuNR rotation data collected at 200 kHz, the angular velocity of *Ec*F_1_ was measured as a function of rotational position during the power stroke in the presence of 1 mM Mg-ATP (Martin et al., 2014). After collecting 5 s of data from each molecule, the power strokes that started at a minimum scattered light intensity at the end of the catalytic dwell (0°) and passed through a maximum intensity upon rotating 90° were collected, and the rotational position *vs.* time for each power stroke was determined for the entire 120° of rotation. This was accomplished using arcsine square root functions to convert light intensity to rotational position (Figure 9A).

Maximum and minimum scattered light intensities were defined as those from the highest and lowest 5th percentile, respectively (Spetzler et al., 2006; Hornung et al., 2011). This aligned the power strokes and minimized phase shift when averaging the data from many power strokes and set the same minimum and maximum intensity values for any one molecule. Average angular velocities (ω) of all power strokes acquired at each rotary position were binned and averaged for every 3° of rotation.

The resulting profile of angular velocity *vs.* degrees of rotation during the F_1_-ATPase power stroke showed that the angular velocity is not constant and undergoes a series of accelerations and decelerations during continuous counterclockwise rotation between catalytic dwells at saturating Mg-ATP (Martin et al., 2014). Due to the low drag imposed on the motor by a 75 × 35 nm AuNR, the velocity changes during the power stroke result from limitations in the rotation rate imposed by the internal mechanism of the motor. The power stroke of *Ec*F_1_ subsequent to the catalytic dwell was divided into two distinct phases. Phase-1 (designated as 0° to 60°) is when ATP binding occurs. Phase-2 (designated as 60° to 120°) is when ADP release occurs.

The small error bars were possible because the angular velocity at each rotary position in Figure 9B is the average of >72,000 power strokes from 22 *Ec*F_1_ molecules (Martin et al., 2014). The designation that the rotary position of the catalytic dwell is 0° resulted from the necessity to align the polarizer with the AuNR prior to data collection. It is important to note that once the power strokes were aligned in this manner for a given F_1_ molecule, the power strokes analyzed at the end of the data set remained aligned with those collected at the beginning at the data set shortly after the polarizer was aligned so that the scattered light from the AuNR was at a minimum during the prior catalytic dwell of the power strokes. The data set of rotation information collected from a given F_1_ molecule includes a total of ~9,000 power strokes of which ~3,000 power strokes were analyzed, which remained aligned with the polarizer the entire duration of data collection. The ability to resolve the changes in angular velocity *vs.* rotational position from data acquired from many F_1_ molecules that were all aligned with the polarizer in the same way clearly demonstrate that the rotary position of the catalytic dwell is a constant from which the power stroke proceeds for 120°.

Closely similar profiles of angular velocity *vs.* degrees of rotation have been observed in power strokes of *Gs*F_1_, and *Ms*F_1_, as well as with *Mm*A_1_ (Sielaff et al., 2016; Ragunathan et al., 2017). Consequently, several steps in the molecular mechanism to drive ATPase-dependent rotation must be shared among this diverse family of motors. It is noteworthy that the angular velocity during Phase-1 is almost 30% slower for *Gs*F_1_ and *Mm*A_1_ than that of *Ec*F_1_. This suggests that the conversion of ATP binding into angular velocity is more efficient for *Ec*F_1_ than for the thermophilic and archaeal rotary motors. Differences in substrate binding affinity and the drag imposed on subunit-γ by the inner surface of the (αβ)_3_-ring may explain these effects. The slower angular velocities of *Gs*F_1_ than observed with *Ec*F_1_ are consistent with the lower torque generated by the former motor.

The F_1_-ATPase has been postulated to operate at 100% efficiency based on the high values of torque (Yasuda et al., 1998; Kinosita et al., 2000, 2004). A consequence of 100% efficiency is that the angular velocity must be constant during the rotation between dwells (Wang and Oster, 1998). The observed accelerations and decelerations during the power stroke provide evidence that the F_1_-ATPase is not 100% efficient at saturating concentrations of ATP.

The angular velocity profile of the F_1_ power stroke during Phase-2 (Martin et al., 2014) correlates well with the trajectory of γ-subunit position derived by targeted molecular dynamics simulations (Pu and Karplus, 2008). This correlation supports the mechanism in which rotation during the final 85° of the power stroke is powered by van der Waals repulsive forces from Mg-ATP binding-dependent movement of the β_E_-lever domain pushing against the γ-subunit as the lever closes. Comparison of catalytic subunit-β conformations show that the lever domain closes upon subunit-γ when ATP binds *via* π–π stacking of the adenine ring with aromatic residues at the base of the β-lever domain and by electrostatic interactions between the nucleotide triphosphates with P-loop residues in the β-catalytic domain. The affinity of the residues on these domains for Mg-ATP decreases the distance between them upon substrate binding.

*Effects of ATP binding on F_1_ power stroke velocity and ATP-binding dwells.* Decreases in ITP-dependent angular velocity observed during Phase-1, a substrate with lower affinity than ATP to *Ec*F_1_, provided the first direct evidence that angular velocity depends on substrate binding affinity (Martin et al., 2014). When *Ec*F_1_ rotation was examined by the AuNR assay, rate-limiting ATP concentrations significantly decreased the average Phase-1 angular velocity between 20°–60°. This decrease resulted from the occurrence of ATP-binding dwells between these rotational positions that approximated a hyperbolic distribution with a maximum at ~36° (Figure 9B **inset**).

The distribution of *Ec*F_1_ ATP-binding dwell positions using the AuNR assay, which provides precise measurements of rotational position, indicates that an ATP-binding dwell can occur over a distribution of rotational positions during Phase-1 of the power stroke that was comparable to the decreases that resulted from ITP binding (Martin et al., 2014). This range of positions fits well with the distribution of *Gs*F_1_ ATP-binding dwell measured using a 40 nm gold bead (Yasuda et al., 2001). It was concluded that ATP-binding dwells occur at ~40° after the catalytic dwell in *Gs*F_1_ under comparable conditions based on measurements in which the centroid position of a ~ 300 nm diameter diffraction limited image from a 40 nm diameter gold bead (Yasuda et al., 2001). The 40° position was the average of the observed distribution, which was thought to result from the error in the measurement. Consequently, it was concluded that at limiting ATP, subunit-γ can rotate beyond 40° without an ATP binding dwell only if ATP has become bound before it reaches that rotational position (the just-in-time binding hypothesis). Otherwise, subunit-γ must wait for ATP to bind before it can rotate beyond 40°. The comparable distribution of the occurrence of *Ec*F_1_ ATP-binding dwells (Martin et al., 2014) conflicts with the just-in-time hypothesis and instead suggest that interruption of rotation during Phase-1 at limiting ATP can occur for a variety of reasons that may be specific to each rotational position between 20°–60°. However, in all cases, the interruption of the power stroke is relieved by the binding of ATP.

The power stroke angular velocity profile *vs.* rotational position of *Mm*A_1_ was not altered by rate-limiting concentrations of ATP and did not give rise to ATP-binding dwells during Phase-1 (Sielaff et al., 2016). Although the V_1_-ATPase from *Enterococcus hirae* V_1_V_O_ (*Eh*V_1_) showed both ATP-binding and catalytic dwells (Iida et al., 2019), the *Paracoccus denitrificans Pd*F_1_, and V/A_1_-ATPase from the bacterial V/A-type ATP synthase of *Thermus thermophilus* (*Tt*V/A_1_) did not exhibit an ATP-binding dwell at rate-limiting ATP concentrations (Furuike et al., 2011; Zarco-Zavala et al., 2020), suggesting that ATP binding of these rotary motors occurs at the same rotary position as the catalytic dwell.

The subunit composition of *Mm*A_1_ and for the *Tt*V_1_ and *Eh*V_1_ is A_3_B_3_DF, where rotor subunits D and F form a helical coiled-coil that extends into the core of the (AB)_3_-ring. The γ-subunit, which serves as the rotor in *Ec*F_1_ and *Gs*F_1_, is comprised of a coiled-coil domain from α-helices that are connected by a globular domain, which may restrict the motion of the rotor relative to that of the A-type rotor. Understanding the differences in sequence and mobility of the F-type and A-type rotors is anticipated to provide important insight concerning the basis for the occurrence and variation in rotary position of ATP-binding dwells.

*Effects of elevated ADP concentrations on EcF_1_ ATPase-dependent rotation.* Elevated ADP concentrations were found to affect *Ec*F_1_ ATPase-dependent rotation in two ways (Martin et al., 2014). First, the presence of ADP suppressed the Phase-1 angular velocity by ~30%, which occurred as the result of an increase in dwells that lasted between 300 μs and 450 μs. These MgADP dwells were maximal at ~36° subsequent to the catalytic dwell (Figure 9C) with a distribution that was comparable to ATP-binding dwells (Figure 9D **inset**). The similarity between *Ec*F_1_ Phase-1 ADP-inhibition dwells and ATP-binding dwells indicates competitive binding of ADP with ATP for β_E_, the empty catalytic site (Martin et al., 2014). Second (Figure 9C), elevated ADP concentrations also decreased the average Phase-2 angular velocity with a distribution of rotary positions between 78° and 110° with a maximum of ~93° where there was also an increase in dwells of comparable duration to those during Phase-1 (Martin et al., 2014). These changes during Phase-2 were proportional to the increase in ADP concentration in solution. This inhibition is consistent with a mass action-dependent decrease in the ability of ADP to be released from the β_D_ catalytic site at elevated ADP concentrations. It is noteworthy that these ADP dependent dwells are unrelated to what is referred to as Mg-ADP inhibition, which occurs during the catalytic dwell and lasts up to 30 s (Hirono-Hara et al., 2001; Sekiya et al., 2010; Bilyard et al., 2013). By comparison, *Mm*A_1_ did not exhibit either ADP-inhibition dwells or show any decreases in the angular velocity of the power stroke during either Phase-1 or Phase-2 in the presence of ADP concentrations as high as 250 μM (Sielaff et al., 2016). These results provide further support for the conclusion that for this A-type ATPase, ATP binds to the β_E_ conformation, and ADP dissociates from β_D_ conformation during the catalytic dwell.

*Energetics of EcF_1_ ATPase-dependent Rotation.* The thermodynamic parameters of the *Ec*F_1_-ATPase power stroke were derived by Årrhenius analysis of the dependence of angular velocity *vs.* rotational position, as a function of temperature, in the presence of saturating Mg-ATP where Δμ_ATP_ = −31.25 k_B_T (Martin et al., 2018). The temperatures examined ranged from 16.3°C to 44.6°C. The *Ec*F_1_-ATPase is believed to remain stable over this temperature range since Årrhenius plots of ensemble ATPase measurements remain linear as high as 55°C. At all temperatures examined, the angular velocity profiles contained a similar pattern of accelerations and decelerations *vs.* rotational position and differed from each other only in the magnitude of angular velocities at various rotary positions.

The angular velocity changed inversely with temperature during most of the first 60° of rotation after the catalytic dwell (Martin et al., 2018). As a result, the activation energy (E_a_) values derived from these Årrhenius plots were negative during Phase-1 of the power stroke and reached a minimum of −3.5 k_B_T after rotation by 34° from the catalytic dwell. As rotation continued beyond 34°, E_a_ values increased to zero at 61°, at which point the angular velocity did not change significantly as a function of temperature (Figure 9D). The E_a_ continued to increase during Phase-2 of the power stroke, reaching the first maximum of 4.3 k_B_T at 79° when subunit-γ rotation was accelerating. At 93°, E_a_ reached a local minimum of 1.3 k_B_T, and then increased again to a maximum of 7.5 k_B_T at 106° during the final deceleration as subunit-γ approached the next catalytic dwell.

Negative E_a_ values indicate that the energy used for work during Phase-1 rotation is of entropic origin, which is characteristic of elastic energy (Mark et al., 1993; Bustamante et al., 1994), and is commonly observed in long biological polymers such as a protein coiled-coil (Wolgemuth and Sun, 2006; Neukirch et al., 2008). Twisting a coiled-coil away from its equilibrium position stores elastic energy that can serve as an entropic spring capable of mechanical work when it unwinds (Panyukov and Rabin, 2000). This is thought to occur because fewer conformations are possible when a coiled-coil is twisted, which reduces the entropy significantly (Treloar, 1975). Allowing a coiled-coil to return to its untwisted equilibrium position exerts a restoring force as the number of possible conformations, and the associated entropy, increase.

The negative E_a_ values during Phase-1 varied with a hyperbolic dependence, indicative of energy derived from the extent of twisting of a coiled-coil from equilibrium (Martin et al., 2018). The axle of the rotary subunit-γ is a long helical coiled-coil that extends through the core of the (αβ)_3_-ring. Assuming that the negative E_a_ values resulted only from compliance of this coiled-coil, the negative hyperbolic E_a_ values were fit to U = ½(κϕ^2^), where U is the amount of stored potential energy as a function of ϕ, the angle of twist of the coiled-coil from its equilibrium position in radians and κ, the spring constant. Using the minimum E_a_ value of 34°, the best fit of the data was achieved with a spring constant of 50 k_B_T•rad^−2^ (205 pN^.^nm^.^rad^−2^). The E_a_ values between 79° and 106° that had a local minimum at 88° were also fit to a plot of energy stored in a torsion spring that fit best with κ = 150 k_B_T•rad^−2^.

It is noteworthy that the inverse of the distribution of the rotary position where ATP-binding dwells occur correlates well with the hyperbolic dependence of the negative E_a_ values observed during Phase-1 (Martin et al., 2018). Likewise, the inverse of the distribution of rotary positions where elevated ADP concentrations suppress angular velocities also correlated with the hyperbolic decrease in E_a_ values during Phase-2 that has a local minimum at 93° (Figures 9C,D). These correlations are consistent with a mechanism in which ATP binds to β_E_ over a range of rotational positions during Phase-1 with a maximal probability of binding at 36°, and where dissociation of ADP from β_E_ occurs over a range of rotational positions during Phase-2 with a maximum probability of ~93°.

The presence of tethers between subunit-γ and the (αβ)_3_-ring that give rise to torsional elastic springs (Saita et al., 2015) have been observed at the same rotary positions as those reported by Årrhenius analysis of the power stroke (Martin et al., 2018). The springs designated I and II were observed at the same rotary positions as those which we reported to occur when ATP bound and ADP dissociated, while the third occurred during the catalytic dwell. These were measured by the forced rotation of a magnetic particle attached to subunit-γ using an external magnet. Although the use of magnetic force to control the rotational position of subunit-γ revealed the existence of these torsional springs, the limits imposed by the magnetic force eliminated the ability to determine the contributions of these springs to the mechanism of the power stroke.

Atomistic simulations of the F_1_ torsional elasticity in conjunction with the (αβ)_3_-ring identified two pair of harmonically coupled subunit-γ coiled-coil segments that had spring constants of 85 and 134 k_B_T•rad^−2^ (Czub and Grubmüller, 2011). These spring constants are similar to 50 and 150 k_B_T•rad^−2^ experimentally observed by Årrhenius analysis of the power stroke (Martin et al., 2018). These γ-coiled-coil segments correspond to the same locations where the β-subunit catch loop and lever domains interact with subunit-γ, consistent with the elastic coupling mechanism.

The free energy of activation (ΔG^‡^) profile of the *Ec*F_1_-ATPase power stroke and its enthalpic (ΔH^‡^) and entropic (TΔS^‡^) components were derived from the Årrhenius analysis (Martin et al., 2018). The enthalpy of activation, which is proportional to E_a_, was also negative during Phase-1. The ΔG^‡^, determined from ΔG^‡^ = ΔH^‡^ − TΔS^‡^, was positive throughout the power stroke because these values were dominated by the entropy of activation (TΔS^‡^).

The thermodynamic values derived for the beginning and end of the power stroke (Martin et al., 2018) are consistent with those values derived for the catalytic dwell from single-molecule measurements of *Ec*F_1_ and *Gs*F_1_ (Sekiya et al., 2010; Adachi et al., 2012; Watanabe et al., 2014). During the catalytic dwell, ATP hydrolysis precedes Pi release, which starts the power stroke. The ΔG^‡^ for ATP hydrolysis from *Gs*F_1_ is comparable to the value at the end of the *Ec*F_1_ power stroke, while the ΔG^‡^ for Pi release in *Gs*F_1_ is only slightly higher than that observed at the start of the *Ec*F_1_ power stroke. Although the ΔG^‡^ values of *Gs*F_1_ for Pi release and ATP hydrolysis correspond to those at the start and end of the *Ec*F_1_ power stroke, the ΔH^‡^ and TΔS^‡^ values from which they were derived differ significantly between the F_1_ from these species. This suggests that the underlying processes that occur during the catalytic dwell and power stroke differ substantially.

The ΔG^‡^ profile *vs.* rotational position is inversely proportional to the angular velocity during the power stroke (Martin et al., 2018). This makes sense because higher angular velocities correspond to lower free energy of activation barriers. The maximum ΔG^‡^ value of 22.6 k_B_T was observed as the power stroke reached 120°, which was the point at which the catalytic dwell began. At all rotational positions, the available energy Δμ_ATP_ (−31.25 k_B_T), which was derived from the ATP/ADP•Pi concentration ratio, was greater than required to overcome the energy barrier of the power stroke (ΔG^‡^) used in the experiments. The efficiency of *Ec*F_1_ calculated from the –ΔG^‡^/Δμ_ATP_ ratio ranged from 62% to a maximum of 72% at 120°. These efficiencies were determined using the AuNR assay under conditions in which the velocity of rotation was not limited by a significant opposing force. Investigations that concluded that *Gs*F_1_ operates with 100% efficiency (Yasuda et al., 1998; Kinosita et al., 2000, 2004) were based on calculations of the ratio of useful work, where “useful work” is defined as the average angular velocity against a rate-limiting opposing force. Operating against a near-stall force is known to increase efficiency of molecular motors including F_1_ (Bustamante et al., 2004).

### The elastic coupling power stroke mechanism of F_1_-ATPase powered rotation

**Figure 10:**
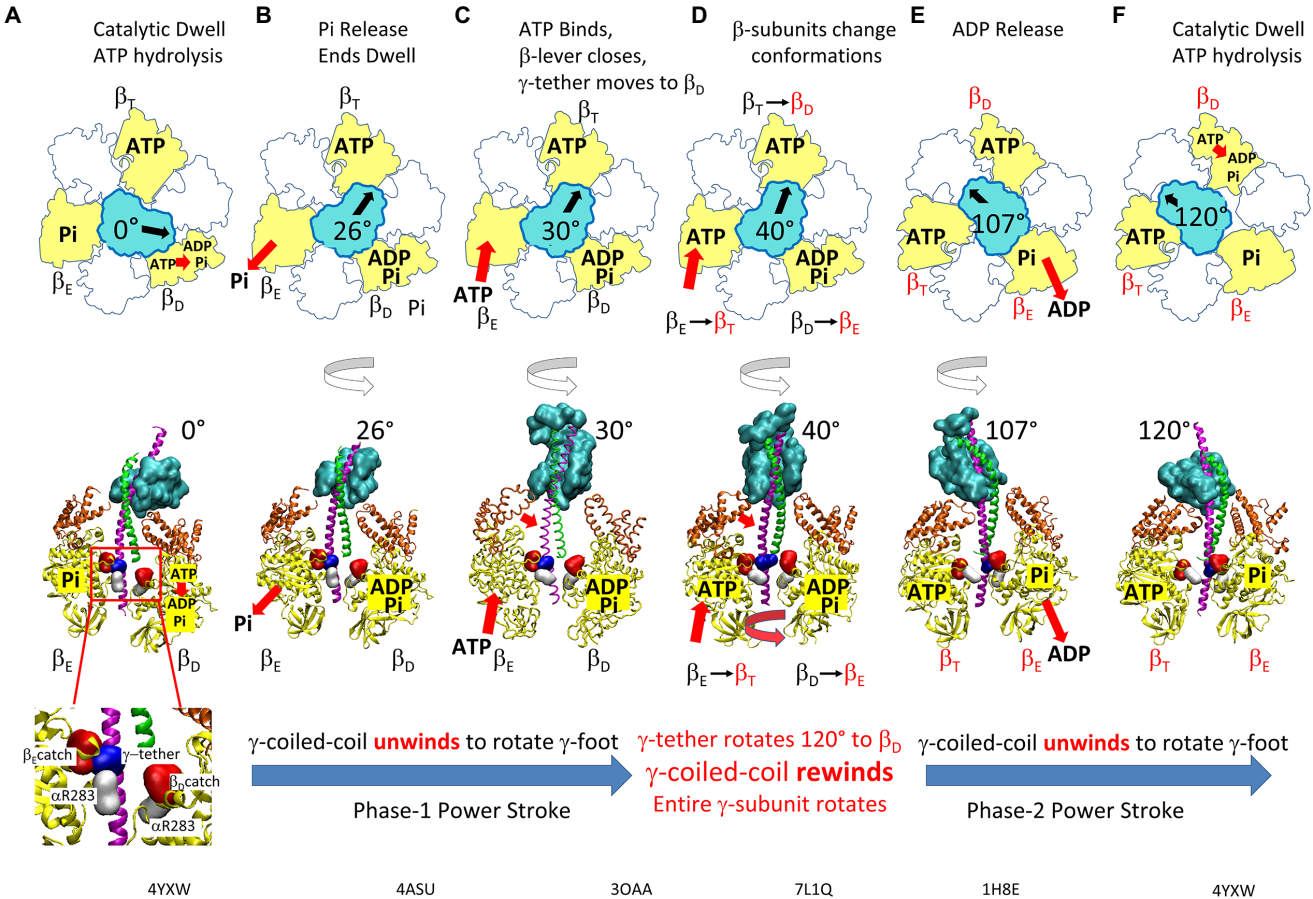
Elastic Coupling Mechanism of the F_1_-ATPase Power Stroke. **(A)** Catalytic dwell where ATP hydrolysis occurs at β_D_. Inset: residues of the β_E_- and β_D_- catch loops (red), the γ-tether (blue), and aR283 (white). **(B)** Pi release from β_D_ initiates Phase-1 power stroke using torsion energy from tightly wound γ-coiled-coil to rotate γ-foot. **(C)** As γ-coiled-coil unwinds, ATP binds β_E_ from 20°–50° (optimally 34°) to force β-lever closed and rotate γ-foot further. **(D)** γ-tether moves from β_E_-catch loop to β_D_-catch to rotate γ-coiled-coil 120° and rewind it to initiate Phase-2 power stroke. This initiates catalytic site conformational changes β_E_ → β_T_, β_T_ → β_D_, and β_D_ → β_E_. **(E)** Release of ADP upon β_D_ → β_E_ occurs from 78°–110° (optimally 93°). **(F)** Catalytic dwell state after γ-foot rotates 120°. Structures corresponding to the indicated γ-foot rotation positions are *Bt*F_1_ 4YXW at 0° **(A)** and 120° **(F)**, *Bt*F_1_ 4ASU at 26° **(B)**, EcF_1_ 3OAA at 30° **(C)**, GsF_1_ 7L1Q at 40°**(D)**, and *Bt*F_1_ 1H8E at 110° **(E)**. Top view structures as viewed from γ-foot, while side view structures omitted α-subunits and the rear β-subunit for clarity. White, α-subunits; yellow, β-subunits; orange, β-levers; cyan, γ-subunit foot; green and pink, γ-coiled-coil. This figure was modified from that in Martin et al. (2018).

The elastic coupling mechanism is based on single-molecule experiments of *Ec*F_1_ ATPase-driven rotation (Martin et al., 2014, 2018), the effects of mutations that altered electrostatic interactions between subunit-γ and the (αβ)_3_-ring (Greene and Frasch, 2003; Boltz and Frasch, 2005, 2006; Lowry and Frasch, 2005), as well as available F_1_ structures including those shown in Figure 10.

During the catalytic dwell at 0° (Figure 10A), the γ–subunit coiled-coil is tightly wound (Martin et al., 2014) where torsion on the coiled-coil is maintained by restraints at both ends. The restraint that is distal from the globular subunit-γ foot domain is composed of the β_E_-catch loop (β_E_D301–D305) and subunit-γ tether (γR268, γQ269) electrostatic interactions (Greene and Frasch, 2003), while the proximal restraint is derived from the closed positions of the six lever domains of the (αβ)_3_-ring (Lowry and Frasch, 2005).

The catalytic dwell ends after ATP hydrolysis at the β_D_-site and Pi release at the β_E_-site (Figure 10B). This opens the β_E_ and β_D_ lever domains and allows the γ-coiled-coil to unwind at the start of Phase-1 of the power stroke. During this phase, which is characterized by negative activation energies (Martin et al., 2018), the β_E_-catch loop remains attached to the subunit-γ tether such that only the subunit-γ foot domain rotates (Figures 10B,C). Rotation is observed because the AuNR is attached to the foot domain.

The binding of ATP can occur during Phase-1 between 20° to 60°, but most commonly at ~36° (Figures 10C,D), which coincides with the rotary position when activation energy reaches a minimum during the power stroke (Martin et al., 2014, 2018). The energy that powers Phase-2 of the power stroke is then derived from ATP binding to β_E_, which depends upon the K_D_ of ATP for the β_E_ catalytic site *vs.* rotary position. The binding of ATP triggers the closure of the β_E_-lever domain and, using van der Waals repulsive forces to push against and rotate the γ-subunit (Pu and Karplus, 2008; Martin et al., 2014), powers Phase-2 of the power stroke (Figure 10E).

ATP binding also induces the transfer of the γ–subunit tether from the β_E_-catch loop to the β_D_-catch loop (Figure 10D). As a result, the γ-coiled-coil rewinds, and the entire γ-subunit rotates during Phase-2. This is a pivotal step in the binding-change mechanism because it induces the conformational changes of the three catalytic sites β_D_ → β_E_, β_E_ → β_T_, and β_T_ → β_D_ (Greene and Frasch, 2003; Martin et al., 2018).

Dissociation of ADP (Figure 10E) occurs during Phase-2 between 78° to 110° with a maximum of ~93° (Martin et al., 2014). At the beginning of the power stroke, this ADP was bound to β_D_. However, due to the catch loop induced β_D_ → β_E_, the dissociating ADP is bound to the newly formed β_E_ that has lower affinity for ADP. Consequently, during Phase-2 of the power stroke, the newly formed β_T_ and β_D_ sites each contain bound ATP, while the newly formed β_E_ site contains bound Pi. In response, the β-lever domains close upon the γ-coiled-coil, trapping it in the twisted, tightly wound conformation that stores torsional energy to be used for the subsequent power stroke (Martin et al., 2018). Consistent with this energy storage mechanism, ATPase activity is severely impacted by mutations that disturb either the interaction between β_E_–catch loop and γ–tether (Greene and Frasch, 2003; Boltz and Frasch, 2006) or that between the β-lever DIIA motif and γ-coiled-coil (Lowry and Frasch, 2005). Disturbing either of these interactions also decreases the torque during a power stroke by about one half (Furuike et al., 2008; Hossain et al., 2008; Usukura et al., 2012).

In all F_1_ structures determined to date, the β_E_–catch loop is engaged with the γ–tether. When structures are aligned using β_E_ residues 5 to 50 of the β-barrel “crown” domain, the difference in rotational position of subunit-γ at the position of the catch loop is minimal. However, the γ–subunit foot domain rotational position varies by as much as 53°, which due to the catch loop-tether attachment, can only happen as the result of unwinding the γ-coiled-coil consistent with the Elastic Coupling Mechanism. Assigning the catalytic dwell at 0° (Figures 10A,F) to ground state *Bt*F_1_ structures 2JDI and 4YXW (Bowler et al., 2007; Bason et al., 2015), unwinding the coiled-coil results in rotation of the subunit-γ foot domain by 26°, 30°, and 40° represented by the Mg^2+^-dissociated structure 4ASU (Figure 10B), the *Ec*F_1_ subunit-ε inhibited *Ec*F_1_ structure 3OAA (Figure 10C), and temperature-sensitive *Gs*F_1_ mutant structure 7L1Q (Figure 10D), respectively (Cingolani and Duncan, 2011; Rees et al., 2012; Sobti et al., 2021), while that of ADP release at 107° (Figure 10E) is represented by pre-product release state *Bt*F_1_ structure 1H8E (Menz et al., 2001).

Although the β_E_-catch loop and γ-tether are attached in all structures, the γ-tether residues are pulled increasingly toward the β_D_-catch loop as the coiled-coil unwinds. Upon rotation of the γ-foot by 40° (*Gs*F_1_ structure 7L1Q), the EcF_1_ equivalent of γR269 has broken free of the β_E_-catch loop now faces the β_D_-Catch loop, while αR283 is positioned to form an electrostatic interaction with the β_E_-Catch loop, validating this mechanism (Greene and Frasch, 2003; Boltz and Frasch, 2006; Martin et al., 2014, 2018). Thus, the exchange of the γ-tether residues from the β_E_-catch loop to the β_D_-catch loop defines the rotational position at which the binding-change conformational changes occur (i.e., β_E_ → β_T_, β_T_ → β_D_, and β_D_ → β_E_).

The rotary position of the γ-foot in *Bt*F_1_ structure 1H8E (Menz et al., 2001), which is 13° CW from structures 2JDI and 4YXW, is consistent with the ADP release occurring late in Phase-2 of the power stroke (Martin et al., 2014). This structure was proposed to be the conformation just prior to release of bound ADP (Menz et al., 2001), since all three catalytic sites contain bound nucleotide and β_E_ contains ADP and sulfate, the Pi analog. Since Phase-2 of the power stroke occurs after the exchange of the γ-tether from the β_E_-catch to the β_D_-catch and its associated conformational changes, the bound ADP and sulfate are in the β_E_-conformation, newly formed after binding-change. It is noteworthy that the other two catalytic sites each contain the transition state analog MgADP•AlF_n_, which suggests that ATP hydrolysis may begin during Phase-2 of the power stroke.

*Elastic Coupling in F_1_F_O_.* The exchange of the subunit-γ tether from the β_E_ catch loop to that of β_D_ was proposed to act as an escapement mechanism to insure tight coupling of substrate binding to the concerted conformational changes during the alternating site mechanism (Greene and Frasch, 2003). The need for an escapement mechanism is of particular importance in F_1_F_O_ to ensure that pmf-driven c-ring rotation only occurs upon ADP and Pi binding to the empty catalytic site. In this mechanism, ADP and Pi binding triggers the release of the β_E_ catch loop, which allows the subunit-γ tether to rotate CW and form electrostatic interactions with β_D_. This in turn changes the conformations of all three catalytic sites to promote ATP release from β_T_.

Measurements of hydrogen/deuterium exchange kinetics of backbone amide groups of F_1_F_O_ support the role of the subunit-γ tether/catch loop interaction as an F_1_F_O_ escapement mechanism (Vahidi et al., 2016). This exchange rate, which depends upon transient H-bond fluctuations in secondary structure, exhibited a pmf-dependent increase specifically in the 16-residue α-helical segment containing the subunit-γ tether residues. The increased exchange rate did not occur in a burst, indicating that the pmf had not caused this α-helical segment to become disordered. Consequently, this is an indication of transient torsional strain that results as the catch loop restrains subunit-γ rotation in F_1_ relative to pmf-driven c-ring rotation in F_O_.

Several cryo-EM F_1_F_O_ structures show the c-ring in multiple rotary positions relative to F_O_ subunit-a in the stator even though the overall rotary position of subunit-γ in F_1_ remains unchanged (Murphy et al., 2019). This is possible because subunit-δ, which attaches the peripheral stalk to F_1_, has flexed to accommodate the differences in rotary positions of the F_O_ and F_1_ rotors. Given the contribution of elastic energy of subunit-γ to the ATPase activity of purified F_1_ (Martin et al., 2018), it is anticipated that the ability of the γ-coiled-coil to twist in F_1_F_O_ would be significantly dampened, which should decrease ATPase activity. However, this remains to be examined. It is noteworthy that the α-helical segment containing the subunit-γ tether to the Catch Loop does show signs of torsional strain in *Sc*F_1_F_O_ structures where the c-ring has rotated by multiple c-subunits relative to the stator (Guo and Rubinstein, 2022) consistent with the results of hydrogen/deuterium exchange kinetics (Vahidi et al., 2016).

### Divergence of F_1_, A_1_ and V/A_1_ ATPase-dependent rotation mechanisms

The power strokes of *Ec*F_1_, *Gs*F_1_, *Ms*F_1_, and *Mm*A_1_ have closely similar profiles of angular velocity *vs.* degrees of rotation (Sielaff et al., 2016; Ragunathan et al., 2017), which support similar rotational mechanisms. The *Ec*F_1_ elastic coupling mechanism (Martin et al., 2014, 2018) has recently been shown to be used by *Gs*F_1_ as well based on cryo-EM structures (Sobti et al., 2021). This includes supporting evidence that ADP dissociates from β_D_ ~ 93° after the catalytic dwell. As a result, the *Gs*F_1_ rotary mechanism has been recently revised (Sobti et al., 2021) to align with that of *Ec*F_1_.

The *Enterococcus hirae* V_1_-ATPase (*Eh*V_1_) showed dwells separated by 40° and 80° rotational steps that appear similar to *Ec*F_1_ and *Gs*F_1_ (Iida et al., 2019). However, the dwell prior to the 40° step of *Eh*V_1_ involves ATP hydrolysis, Pi release and ATP binding, while the dwell prior to the 80° step involves release of ADP. *Paracoccus denitrificans Pd*F_1_ (Zarco-Zavala et al., 2020), *Tt*V/A_1_-ATPase (Furuike et al., 2011), and *Mm*A_1_ (Sielaff et al., 2016) did not give rise to ATP-binding dwells separate from the catalytic dwells. This suggests that the rotary position for ATP binding to these rotary motors occurs at the same rotary position as that at which ATP hydrolysis and Pi release occur. The rotary position of the ATP-binding dwell of *Homo sapiens* F_1_ (*Hs*F_1_) and *Bt*F_1_ occurs 30° and 40° after the catalytic dwell, respectively, and Pi is released 95° after the catalytic dwell (Suzuki et al., 2014; Kobayashi et al., 2020). The underlying mechanistic reasons that the ATP synthases from these organisms differ in the rotational positions for these steps are currently not understood.

## Single-molecule rotation of F_1_F_O_ embedded in lipid bilayer nanodiscs

**Figure 11:**
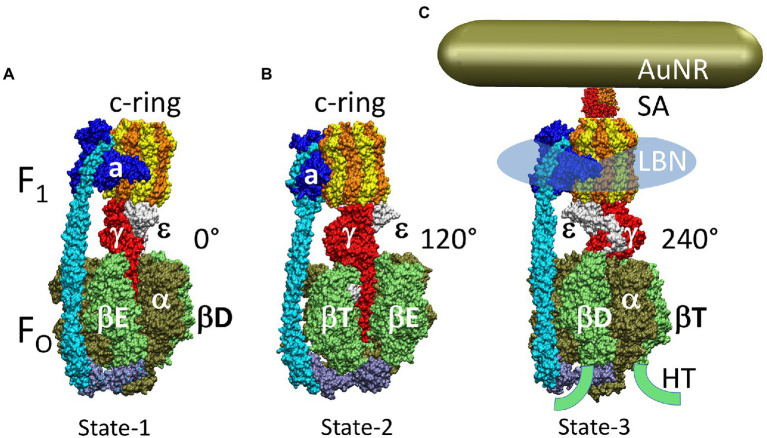
Cryo-EM structures of the three rotary states of *Ec*F_1_F_O_ ATP synthase inhibited by ADP. **(A)** State-1 *E*cF_1_F_O_ structure 6OQU. **(B)** State-2, *E*cF_1_F_O_ structure 6OQV with rotor 120° CCW from A where subunit-α is not shown to reveal subunit–γ. **(C)** State-3 *E*cF_1_F_O_ structure 6WNR with rotor 240° CCW from A showing microscope slide assembly of F_1_F_O_ embedded in a lipid bilayer nanodisk (LBN) for rotation measurements. His_6_-tags (HT) on β-subunit C-termini enabled attachment to slide, while the AuNR coated with streptavidin (SA) bound to the biotinylated subunit-c ring. This figure was modified from Yanagisawa and Frasch, (2021).

Single-molecule studies have been difficult for membrane-bound molecular motors like F_1_F_O_ where proton transport across a lipid bilayer is used to synthesize ATP. These problems were circumvented by embedding the F_O_ portion of *Ec*F_1_F_O_ into a lipid bilayer nanodisc that has been shown to provide a good model for lipid bilayers (Ishmukhametov et al., 2010). The nanodisc are large enough to allow the incorporation of the F_O_ complex and a few hundred lipid molecules yet are on the same scale as the F_1_F_O_ complex, which is required for single-molecule studies (Figure 11C).

Assembly of stable nanodisc-F_1_F_O_ complexes from membrane scaffold protein (MSP) and detergent-solubilized ATP synthase was verified by 2D electrophoresis where the first nondenaturing gel dimension contained one prominent band (Ishmukhametov et al., 2010). This band contained both MSP and all F_1_F_O_ subunits when separated in the second denaturing gel dimension. When F_1_F_O_ was incorporated into nanodisc, the initial ATPase activity after purification was 1.5-fold higher than the detergent solubilized protein and did not decline significantly after the preparation had been at 25°C for 8 h. DCCD inhibited ATPase activity by 85%, indicating that there was strong coupling between hydrolysis and proton transport. By comparison, ATPase activity of detergent solubilized F_1_F_O_ lost all activity and aggregated within a few hours at room temperature.

The mutation c2∇C was made to the c-subunit, which inserted a cysteine residue at position-2 in the c-subunit to a cys-free *Ec*F_1_F_O_ that was used for biotinylation (Ishmukhametov et al., 2010). An AuNR was then attached to the ten biotins on the c-ring after the nanodisc-F_1_F_O_ complexes (hereafter F_1_F_O_) were attached to the cover slip *via* his-tags at the F_1_ β-subunit N-terminus for single-molecule studies to examine ATPase-driven rotation when scattered red light intensity was acquired at 5 μsec intervals. ATPase-dependent rotation of F_1_F_O_ molecules was measured at 1 mM Mg-ATP, 50 μM ADP, and 50 μM Pi at pH 8.0 (Ishmukhametov et al., 2010). This saturating concentration of ATP was used because under these conditions, F_1_-ATPase-driven rotation events occur in continuous 120° power strokes between catalytic dwells that are not interrupted by ATP-binding dwells. Prior to data collection, the polarizer was rotated to align the AuNR so that the scattered red light intensity was at a minimum at one of the three catalytic dwells and the intensity of the subsequent power stroke increased through a maximum scattered light intensity upon rotation at 90°. Scattered light intensity *vs.* time was then collected from the AuNR attached to a given F_1_F_O_ molecule for 50 s during which ~3,520 power stroke events were monitored. The rotational position during each power stroke was determined *vs.* time using *Eq. 1*.

**Figure 12:**
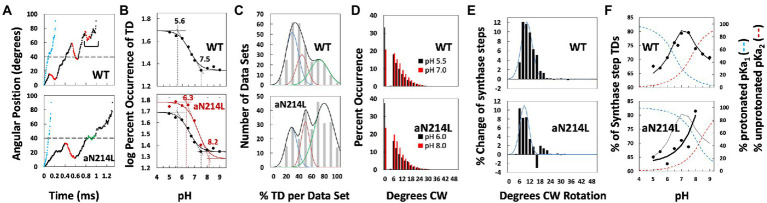
Characterization of F_O_ transient dwells (TDs) and ATP-synthase direction steps in WT and the subunit-a N214L mutant. **(A)** Example F_1_F_O_ ATPase-dependent power strokes without TDs (blue), and power strokes with TDs that contain (red) or lack (green) ATP synthase direction c-ring rotation relative to subunit-a plotted as degrees of rotation where 0° is the catalytic dwell and ATP-binding and/or ADP-inhibition occurs at ~35°. Brackets indicate Brownian-type oscillations during a TD. **(B)** Average TDs per data set vs. pH showing pKa values for WT (black) and aN214L (red). **(C)**. Distributions at pH 6.0 of the percent of TDs per power stroke data set (gray bars) where each data set contained ~300 power strokes that were collected from ~50 different F_1_F_O_ molecules, and data were binned in 10% increments. The data were fit to the sum of three Gaussians (black line) representing low (blue), medium (orange), and high (green) TD formation efficiencies. **(D)** Distribution of the extent of CW synthase-direction rotation during TDs at the low and high pH values where the percent of synthase-direction steps was maximum and minimum. **(E)** Gaussian distributions (blue) of the difference between low and high pH values from **D** in extent of CW synthase-direction step rotation. **(F)** Percent of TDs containing CW synthase-direction steps vs. pH where data were fit to the sum of Henderson–Hasselbalch curves (black) resulting from the fraction of protonated groups with pKa_1_ (blue) and unprotonated groups with pKa_2_ (red) vs. pH. This figure was modified from Yanagisawa and Frasch, (2021).

Two populations of F_1_F_O_ power strokes were observed (Ishmukhametov et al., 2010). The power strokes of one population rotated continuously comparable to that observed with purified F_1_-ATPase-driven CCW rotation, which required ~200 μs to rotate 90° (Figure 12A). The other population of molecules took much longer to complete a power stroke, due to the appearance of what were designated transient dwells (TDs). Since the average transient dwell duration was ~150 μs, the total time required to rotate 90° in the presence of transient dwells was ~650 μs. Under these conditions, TDs were present in ~22% of power strokes examined, of which about 70% were able to rotate the c-ring in the CW direction against the torque generated by the F_1_ power stroke. The extent of these ATP synthase direction rotations during these dwells averaged 11° and did not exceed 36° (Yanagisawa and Frasch, 2017, 2021).

Due to the sinusoidal dependence of scattered light intensity *vs.* rotational position, the first 90° of each power stroke was examined for the presence of TDs when light intensity increased from a minimum through a maximum. For power strokes that contained TDs, an average of ~2.5 TDs occurred during the 90° of rotation examined. Measurements of both the rotational spacing of TDs and the average number of TDs translate to ~10 transient dwells for each complete 360° revolution of the c_10_-ring, or one TD per c-subunit. This indicated that TDs result from an interaction between subunit-a and each successive c-subunit during rotation. These data also showed that rotational stepping events that skip one or two c-subunits were a rare occurrence.

Transient dwells were eliminated from F_1_F_O_ power strokes by a mutation to subunit-a (a∇14) that inserted 14 amino acids into subunit-a (Ishmukhametov et al., 2010). This mutation, which did not alter F_1_F_O_ assembly, subunit composition or ATPase activity, was not susceptible to DCCD inhibition, and inverted membranes containing this mutant were unable to catalyze ATPase-dependent proton translocation. The results from this mutation indicated that TDs arise from the periodic interaction between subunit-a and each successive c-subunit in the c-ring.

### Viscosity dependence of transient dwell formation

Increasing the drag on the AuNR by changing the viscosity from 0.9 cP (without PEG400) to 1.8 cP (15% PEG400) did not decrease the average angular velocity of the F_1_F_O_ ATPase-dependent power strokes (Ishmukhametov et al., 2010) as was also observed with purified F_1_ (Spetzler et al., 2009). However, concurrent with the viscosity-dependent decrease in average angular velocity between 1.8 cP and 4.3 cP, the abundance of TD-containing power strokes increased from 27 to >80%. At viscosities that exceeded 7.5 cP, the angular velocity of the F_1_F_O_-ATPase-dependent power strokes were too slow to observe a difference in the rate caused by the presence of TDs. In contrast, none of the power strokes from F_1_F_O_ molecules containing the subunit-a∇14 insert exhibited transient dwells regardless of the PEG400 concentration.

To observe the distribution of F_1_F_O_ power strokes that contain TDs, rotation data from each molecule was collected for a total of 50 s in ten 5s data sets and the percent of power strokes containing TDs were binned into 10% increments (Martin et al., 2015). Data sets were collected from ~50 molecules, which allowed the distribution of the percent of TDs per data set to be determined. These distributions showed the same average increase in the percent of TDs *vs.* viscosity observed by that obtained from the average of all power strokes. At a viscosity of 4.3 cP, the distribution of TDs per data set had a single maximum of ~85% with very few data sets of <50%. The distribution of the cD61G mutant was clearly bifurcated with local maxima of ~20 and 90%. This suggested that at high viscosities, TDs can originate from the proton translocation-dependent process involving c-ring rotation, and by a second interaction that was independent of proton translocation. Similar results were observed with mutations cR210G and cR210L that removed the guanidinium group thought to displace the proton into the output channel of subunit-a.

The highly conserved subunit-a residue aE196 was identified as a participant in output channel proton translocation based on observations that aE196Q and aE196L mutations: (1) showed similar decreases in TDs per data set to that of cD61G at elevated viscosity where the distribution was bifurcated into local maxima of 30 and 60%; (2) decreased ATP synthase activity by 11- and 15-fold; and (3) increased NADH-dependent ACMA quenching in inverted *E. coli* membrane vesicles similar to the effects of cD61G. This was the first residue to be identified as a component of an F_O_ proton output channel (Martin et al., 2015).

The average transient dwell duration was ~200 μs at 1.8 cP, which decreased to ~50 μs at 5.5 cP. The decrease in dwell duration was compensated by slower power stroke velocities at high PEG400 concentrations such that the power stroke velocity was too slow to distinguish changes in velocity that resulted from the existence of TDs (Ishmukhametov et al., 2010).

The increase in the abundance of TD-containing power strokes at pH 8.0 and viscosities >1.8 cP *vs.* PEG400 correlated with the subset of *Ec*F_1_F_O_ molecules subjected to loads exceeding ~1.4 pN ms as the result of the drag on the AuNR (Ishmukhametov et al., 2010). Molecules subject to drag exceeding the 1.4 pN ms threshold had power stroke angular velocities observed at viscosities between 1.8 and 5.5 cP, which corresponded to molecules with velocities less than 220° ms^−1^. Since the interaction responsible for TD formation occurs every ~36° (i.e., between subunit-a and each c-subunit), the time constant for TD formation is ~160 μs. Thus, any molecule that rotates 36° in <163 μs does not exhibit transient dwells. Given the dependence of TD duration on viscosity, these results indicate that the time constant for the termination of the interaction is ~175 μs, which is independent of viscosity, and the turnover time for the interaction between subunit-a and subunit-c is then ~338 μs.

At the slowest angular velocities of any given molecule, the subunit-a/subunit-c interaction forms after the c-ring rotated CCW only a few degrees. This interaction between the a/c subunits must then act as a leash that allows rotation to continue to a limit of ~36° after it forms (Ishmukhametov et al., 2010). The extent to which the c-ring rotates CCW after the 163 μs interaction formation time decreases as the angular velocity is slowed by increased load on the motor. For example, at viscosities between 3.0 cP and 5.5 cP, the interaction forms after the c-ring rotated ~31° and 21°, respectively. It is noteworthy that these time constants are specific for the viscosity-dependent interaction between subunit-a and each c-subunit in the c-ring, which were measured at pH 8.0 where there was evidence that the leash interaction had a proton-translocation-dependent component, and a proton-translocation independent component (Ishmukhametov et al., 2010; Martin et al., 2015). The viscosity of the aqueous spaces inside mitochondria is comparable to that which gives rise to the high occurrence of TDs that we observed. Of course, the F_1_F_O_ inside mitochondria are not attached to an AuNR that slows c-ring rotation as the result of drag at these viscosities. However, it is possible that PEG400 may also affect rotation by dehydrating the protein. Dehydration could enhance the strength of hydrophobic subunit interactions that could contribute to the proton translocation independent leash, and slow the rate that protons enter and leave the subunit-a half-channels, which are necessary for proton-translocation-dependent TD formation.

#### pH dependent transient dwells and synthase-direction steps

The pH dependence of TD formation was examined in the absence of PEG400 (Yanagisawa and Frasch, 2017). The average occurrence of ~22% TDs per data set that was previously observed at pH 8.0 (Martin et al., 2015) was observed between pH 9.0 and pH 7.5 (Yanagisawa and Frasch, 2017). From pH 7.0 to pH 5.0, the percent of TDs increased to an average occurrence of ~50% at pH 5.0. This pH dependence was fit to a Henderson-Hasselbalch relationship, which indicated that the observed TDs resulted from proton-translocation-dependent events, which suggested that protonation of a group with a pKa of 6.3 was necessary to form a TD. However, the average changes in TD formation between pH values 6.0 and 7.5 did not fit well to the dependence on the protonation of a single group.

The TDs observed *vs.* pH during F_1_F_O_-ATPase-dependent power strokes share many similarities with those formed as the result of high viscosity (Yanagisawa and Frasch, 2017). TDs occur with an average periodicity of every ~36° throughout the power stroke, which did not change between pH 5.0 and 9.0 (Figure 12A) consistent with successive stepping of single c-subunits of the *E. coli* c_10_-ring relative to subunit-a. The TDs also either stopped CCW rotation, or induced CW rotation against the force of the ATPase power stroke (synthase direction steps) (Figure 12A).

Differences between pH-dependent TDs (Yanagisawa and Frasch, 2017) and viscosity-dependent TDs (Martin et al., 2015) include the dwell duration time and the fraction of TDs that undergo synthase direction steps. The duration of proton-translocation-dependent TDs reached a maximum of ~158 μs at pH 8.0 and was at a minimum of ~118 μs at both pH 5.0 and pH 9.0. By contrast, the viscosity-dependent TD dwell durations at pH 8.0 decreased *vs.* viscosity from ~158 μs in aqueous medium to ~50 μs at viscosities of 5.5 cP. Importantly, the fraction of TDs that contained a synthase-direction step varied as a function of pH, with a maximum of 80% at ~pH 7.3, and minimum values of 67 and 71% at pH values of 5.5, and > 7.5, respectively. By contrast, the fraction of TDs containing synthase steps of ~70% in aqueous buffer at pH 8.0 did not change with viscosity.

*Synthase steps can be Power strokes or Brownian Ratchets.* A power stroke mechanism has been defined as the generation of a large free energy gradient over a distance comparable to the step size of the molecular motion so that transition to the forward position occurs nearly irreversibly (Hwang and Karplus, 2019). By contrast, in a Brownian ratchet mechanism the motor is thought to visit previous and forward positions through thermal motion, where stabilization in the forward position results in conformational changes triggered by the fuel processing event. Some synthase-direction steps rotated CW in a concerted, and apparently irreversible manner characteristic of a power stroke (Figure 12A), while others, as indicated by brackets in Figure 12A were observed to oscillate back and forth during the TD (Yanagisawa and Frasch, 2021). The TDs with oscillations most commonly occur late in the F_1_ power stroke (~70–80°) and were more pronounced in all mutations examined except aN214L. Such oscillations are direct evidence of a Brownian ratchet mechanism and are likely the result of a close balance between the energy that powers the F_1_-ATPase power stroke with the energy that powers synthase-direction rotation, which suggests that these mutations cause a decrease in the energy to power synthase-direction rotation.

*TD formation efficiencies correlate with EcF_1_F_O_ structural states.* The distribution of the percent of TDs per data set determined at each pH did not fit to a single Gaussian curve (Yanagisawa and Frasch, 2017). These distributions had similarities to those obtained *vs.* viscosity. While the viscosity-dependent TD distributions were based on data from 25 F_1_F_O_ molecules (~75,000 power strokes) at each viscosity, data was collected from ~50 F_1_F_O_ molecules (~150,000 power strokes) at each pH to increase the resolution (Figure 12C). Good fits of the distributions at each pH fit were achieved by the sum of three Gaussian curves where the probability of forming transient dwells was low, medium, and high. The average values of TDs represented by each Gaussian curve increased inversely with pH. The Gaussian distribution with the highest probability of forming TDs increased by the greatest extent with an average value of 70% TDs per data set at pH 5.0. In several data sets, TDs were present in 100% of the power strokes.

Single particle cryo-EM structures of *Ec*F_1_F_O_ revealed three unique states that differed by the asymmetric rotary positions of central stalk subunits –γ and –ε relative to subunit-a and the peripheral stalk (Sobti et al., 2016). These are equivalent to the rotary positions of the drive shaft between the 120° ATPase-driven power strokes (Figures 11A–C). For each F_1_F_O_ molecule examined (Yanagisawa and Frasch, 2017), only one of the three consecutive power strokes, which together comprise a complete 360° rotation, was analyzed for the presence of TDs. Thus, for every 51 F_1_F_O_ molecules analyzed, it is likely that the power strokes specific to each of the three structural states was sampled by 17 molecules.

Due to asymmetry in *Ec*F_1_F_O_ between the three F_1_ catalytic sites that rotate the γ–subunit axle in 120° power strokes with the F_O_ c_10_-ring that rotates in 36° steps concurrent with the transport of each proton, the c-ring will rotate 4 × 36° for one power stroke, and 3 × 36° for the other two. The positive and negative torsion on the c_10_-ring from the elastic energy needed to accommodate the +14° and −14° misalignments between F_1_ and F_O_ during two of the three power strokes was proposed to be responsible for the differences between the high and low probabilities of forming TDs relative to the medium TD-forming probability when the c-ring and the F_1_ catalytic dwells are aligned (Yanagisawa and Frasch, 2017).

The results of the single-molecule AuNR *Ec*F_1_F_O_ rotation studies, which showed the high, medium and low probabilities of TD formation, were compared to other single-molecule analyses of fluorescence video-microscopy of *Ec*F_1_F_O_ rotation. For instance, analyses that used actin filaments, those that used Fōrster resonance energy transfer between pairs of fluorescent probes attached to the *Ec*F_1_F_O_ stator and rotor that give rise to low, medium, and high FRET efficiencies, etc. (Sielaff et al., 2019). All three assays demonstrated differences among the three consecutive 120° power strokes that support the conclusion that the +14° and −14° misalignments between F_1_ and F_O_ in two of the three structural states of the complex is compensated by internal elastic coupling between F_1_ and F_O_, which is responsible for the differences in efficiencies of the three rotary steps. The three structural states of *Ec*F_1_F_O_ determined by cryo-EM differ in the rotary position of the c_10_-ring relative to subunit-a by 108°, 108°, and 144° consistent with 3 protons translocated during two ATPase power strokes and 4 protons translocated during the third (Sobti et al., 2016). As a result of the asymmetric binding of subunits γ and ε to the c-ring, each c-subunit is unique such that aR210 is positioned adjacent to cD61 residues of adjacent c-subunits chains M and N in state 3 (*Ec*F_1_F_O_ structure 5 T40), chains S and T in state 2 (*Ec*F_1_F_O_ structure 5T4P), and chains P and Q in state 3 (*Ec*F_1_F_O_ structure 5T4Q). During power stroke-1, c-subunits M, V, U, T rotate past subunit-a thereby translocating 4 protons, while in power stroke-2 and power stroke-3, c-subunits S, R, Q, and subsequently P, O, N rotate past subunit-a that each translocate 3 protons.

Each power stroke is also unique because a portion of subunit-γ must pass through the narrow gap created by the peripheral stalk during power stroke-1, and a portion of subunit-ε must pass through this gap during power stroke-3. However, power stroke-2 is not affected in this manner (Sobti et al., 2016). As the result of these structural differences, we postulated that the low, medium, and high probabilities of forming TDs using the AuNR rotation assay result from rotational transitions from structure 5T4O to 5T4P, from structure 5T4P to 5T4Q, and from structure 5T4Q to 5T4O, respectively (Sielaff et al., 2019).

*pKa Values of Transient Dwells.* The F_1_F_O_ cryo-EM structures show that two c-subunits from the c-ring are in contact with subunit-a at a time (Sobti et al., 2016). During CW rotation in the ATP synthesis direction, the leading c-subunit must accept a proton from the input channel while the lagging c-subunit donates its proton to the output channel. The poor fit of the pH dependence of TD formation to a single Henderson-Hasselbalch equation for protonation of a group with a single pKa value suggested that this pH dependence resulted from both the protonation and deprotonation events that operated with different pKa values (Yanagisawa and Frasch, 2021).

Equations were used that define the pH dependence of enzyme inhibition kinetics (Cook and Cleland, 2007) to determine the pKa values precisely (Yanagisawa and Frasch, 2021). A TD occurs when subunit-a binds to the c-ring to stop F_1_ ATPase-driven rotation temporarily, which can happen 3.6 times per F_1_ power stroke. Kinetically, the duration of a 120° without TDs is ~260 μs, and the duration of a power stroke containing 3.6 TDs is ~720 μs, which represents a 64% decrease (inhibition) in the rotation rate. The average occurrence of TD formation for the three efficiencies of TD formation *vs.* pH were fit to these equations to determine the pKa values (Figure 12B). The average maximum value of 47.5% TD occurrence occurred at pH 5.0, which decreased with increasing pH until it plateaued at a minimum of ~22% at pH values >7.5. Observation of such a minimum value with increasing pH indicated that a TD can form as the result of the presence of an unprotonated group with a high pKa, but that the efficiency of TD formation increases with decreasing pH when a second group with a low pKa value becomes protonated. The equation that defines this relationship is based on a log function. As a result, when plotted as the log of the percent of TDs formed versus pH, the two pKa values were determined by the intersection of the pH-dependent increase in TD formation with the high and low plateaus (Figure 12B). In this manner, the pKa values of the groups that must be protonated and unprotonated to induce a TD were 5.6 and 7.5, respectively.

The proportions of TDs with synthase-direction steps depends on the proportion of both the high pKa and low pKa groups in the correct protonation state to enable proton transfer to and from the carboxyl groups of the leading and lagging c-subunits in the ring, respectively (Yanagisawa and Frasch, 2021). The subset of TDs that forced the c-ring to rotate CW (synthase-direction step) against the CCW force of F_1_-ATPase rotation had a maximum of 80% at ~pH 7.3 and a minimum of 67% at pH 5.5. At pH values >7.5, the proportion of synthase-directions steps also decreased to 71% at pH 9.0. The pH dependencies of TDs with and without synthase-steps fit well to curves calculated from the proportions of the group with low and high pKa values that were protonated and unprotonated, respectively. Consequently, TDs that lacked a synthase-direction step reached a maximum of the low and high pH values examined when only the group with pKa_1_ or with pK_2_ were protonated and unprotonated, respectively. Conversely, the pH-dependence of TDs containing synthase-direction steps required the correct protonation from the groups with both high and low pKa values to enable proton transfer to and from the c-ring (Figure 12F).

*Subunit-a mutations from either half-channel alter both high and low pKa values. E. coli* subunit-a residues aN214, aE219, aH245, aQ252 of the synthase-direction proton input channel and aE196 of the synthase-direction output channel were initially identified by site-directed mutagenesis studies by their impact on ensemble assays of ATP synthesis, ATP hydrolysis, and ATPase-dependent proton pumping activity. (Lightowlers et al., 1988; Vik et al., 1988; Howitt et al., 1990; Eya et al., 1991; Hartzog and Cain, 1994; Hatch et al., 1995; Fillingame and Steed, 2014; Martin et al., 2015). These groups were originally thought to function by directly transferring protons from one group to the next. Cryo-EM F_1_F_O_ structures that revealed details of subunit-a confirmed that these residues are positioned along possible half-channels that are separated by aR210 (Martin et al., 2015; Zhou et al., 2015; Hahn et al., 2018; Pinke et al., 2020; Sobti et al., 2020) where the role of aR210 has been thought to be responsible for deprotonation of the proton from the lagging c-subunit carboxyl group sending the proton to the output channel. Alternatively, proton translocation through F_O_ has been postulated to occur *via* a Grotthuss mechanism where the input and output channel residues are hydrogen-bonded to a column of single water molecules that behave in a coherent manner to transfer protonic charge over long distances *via* rapid exchange of protons between H_3_O and H_2_O (Feniouk et al., 2004; Cukierman, 2006).

It was anticipated that if the subunit-a channel residues were directly transferring protons, then mutation of any of the known channel residues would impact the pKa of only the half-channel in which it is located. However, mutations aN214L, aQ252L, aE219L, aH245L, and aE196L each changed both the pKa values of both half-channels. For example, aN214L changed both the low and high pKa values associated with proton transfer to and from, respectively the c-ring (Figure 12B). This also changed the pH dependence of synthase-direction steps (Figure 12F), which depend on the sum of the proportion of protonated input channel residues with the low pKa, and the proportion of unprotonated output channel residues with the high pKa. Note that these pKa values do not represent a single amino acid sidechain, but the average of pKa’s for the entire channel. In addition, mutation of any single residue did not completely eliminate the proton translocation-dependent synthase-direction steps.

These results strongly support a Grotthuss mechanism in F_O_ where simultaneous stepwise movement of protons distributed along water columns in the two half-channels communicate *via* rotation-dependent proton transfer to and from the leading and lagging c-ring cD61 carboxyls *in lieu of* transferring protons directly and independently (Yanagisawa and Frasch, 2021). In *E. coli*, the coherent behavior of the water columns enables the release of a proton to the cytoplasm concurrent from the output channel with each proton that enters the subunit-a *via* the input channel from the periplasm. This conclusion is also consistent with the fact that participating residues aS199, aN214, and aQ252 are polar but not ionizable, and distances between channel residues are too far apart for direct proton transfer but are positioned at distances able to support a water column. Although aQ252 is highly conserved, glycine or hydrophobic groups naturally occur at positions aN214 (e.g., *T. gondii, and T. thermophilus*), for aH245 (e.g., *M. phlei, T. thermophila, A. woodiii, T. gondii, I. tartaricus, F. nucleatum, and T. thermophilus*) and for aE219 (e. g. *T. thermophila, E. gracilis, B. pseudofirmus OF4, P. angusta, S. cerevisiae*, and *A. platensis PCC9438*). Such mutations can be tolerated if the primary role of these groups is to support a water column that transfers protons.

Recent *Polytomella*F_1_F_O_ and *Bt*F_1_F_O_ structures were of sufficient resolution to observe density near the input channel residues consistent with Grotthuss-type water molecules in this half-channel (Murphy et al., 2019; Spikes et al., 2020). Unidentified electron densities near subunit-a input channel residues in *Ec*F_1_F_O_ structures also suggest the presence of bound waters (Sobti et al., 2020). The observation of a water column in both half-channels of the V_O_ complex (Roh et al., 2020) suggests that Grotthuss-based proton translocation is a commonly shared trait among the greater family of rotary ATPases.

**Figure 13:**
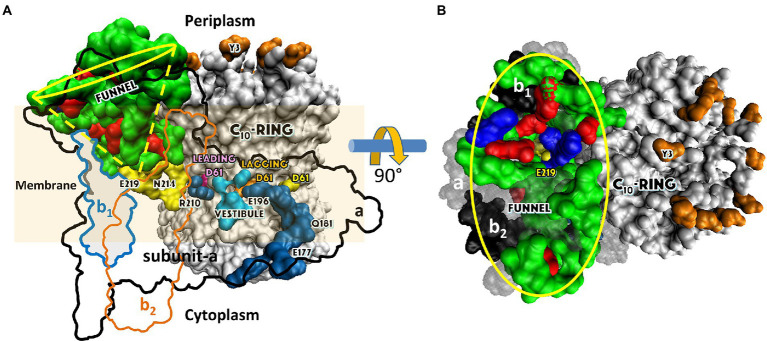
Aqueous Funnel of charged and polar groups can serve as an antenna to supply protons to the input channel. **(A)** Transmembrane view of *E. coli* F_O_ (pdb-ID 6OQR) showing the path of charged and polar residues across the membrane. Outlines indicate space occupied by hydrophobic residues in subunit-a (black line), subunit-b_1_ (blue line), and subunit-b_2_ (orange line). The inner surface of the Funnel, which is lined with polar residues and loop regions (green), acidic groups (red) and histidines (blue) from subunit-a and the subunit-b_1_ N-terminus, is exposed to the periplasm at its wide end that narrows to aE219 (yellow) at the bottom. The input channel (yellow) extends from aE219 to aN214 and aQ252, which are proximal to aR210. Between aR210 and the Output Channel (dark blue) the leading (pink) and lagging (orange) cD61 groups rotate through a Vestibule lined above and below the cD61 rotation plane by polar sidechains (light blue) that decrease the dielectric constant of the vestibule from that of the lipid bilayer. A protonated cD61 exposed to the lipid bilayer (yellow) is also visible. **(B)** Periplasmic surface of F_O_ showing the interior surface of the Funnel (yellow oval) lined with charged and polar groups from subunit-a and subunit-b_1_ as in **(A)** that narrows to aE219 (yellow) at the bottom where the input channel begins. Hydrophobic residues are shown of subunit-a (gray), subunits-b_1_ and b_2_ (black), and the c-ring (white). The cY3 sidechains (orange) are shown to indicate the orientation of the periplasmic surface of the c-ring. This figure was modified from Yanagisawa and Frasch, (2021).

A Grotthuss mechanism was first proposed to explain extremely high rates of F_O_-dependent proton translocation across *R. capsulatus* membranes (Feniouk et al., 2004). The rates were so fast that an ~40 Å diameter Coulomb cage lined with charged and polar groups was proposed to be required to serve as a proton antenna to increase the delivery rated of protons from the aqueous solution to the entrance of the input channel water column (Wraight, 2006). A ~ 30 Å diameter funnel (Figure 13) lined with carboxylate and imidazole residues as the funnel narrows has been identified in *Ec*F_O_ (Yanagisawa and Frasch, 2021) where aE219, which was proposed to be the start of the Grotthuss column, is positioned at the apex of this funnel.

*Proton translocation ATP synthase-direction steps rotate the c-ring 11° CW.* The proportion of TDs with and without a synthase-direction step varied as a function of the pH (Figure 12D). In WT and aN214L, the minimum proportion of synthase-direction steps (black bars) was observed at the lower end of the pH scale of pH 5.5 and 6.0, respectively. Even at these low pH values, synthase-direction steps accounted for ~67% of all TDs in WT. The maximum proportion of synthase-direction steps (red bars) was observed at a neutral pH of 7.0 for WT and 8.0 for aN214L. In WT, a maximum of ~80% of TDs contained synthase-direction steps at pH 7.0, which was an increase of 13% from the minimum. (Yanagisawa and Frasch, 2017, 2021).

After subtracting the occurrence of the extent of synthase-direction step CW rotation at the pH when it was at a minimum (black bars) from that observed at its maximum (red bars), a Gaussian distribution of the increase in the extent of synthase-direction step CW rotation was observed (Figure 12E). These plots show the distributions of the extent of CW rotation during a synthase-direction step, for which the 11° ± 3 average values of CW rotation were not changed significantly by the mutations (Figure 12E). However, they did alter the pH dependence of the occurrence of synthase-direction steps in a manner that showed that these steps involved protonation and deprotonation of the leading and lagging c-subunits, respectively, of the c-ring (Figures 12B,F). The effects of the subunit-a mutations on the synthase-direction steps ruled out the possibility that these steps result from twisting the entire F_O_ relative to F_1_. During a CCW F_1_-ATPase power stroke, TDs occur every 36°, which is equivalent to an interaction between subunit-a and each successive c-subunit in the *E. coli* c_10_-ring. Since synthase-direction steps rotate by 11°, rotation by an additional 25° is required to advance the c-ring by one full c-subunit, which we observed in only 0.1% of the synthase-direction steps. Rotational sub-state structures (pdb-ID 6OQR and 6OQS) of ADP-inhibited *E. coli* F_1_F_O_ that differ by a 25° rotation of the c-ring relative to subunit-a were obtained by cryo-EM (Sobti et al., 2020). Since advancing the c-ring by one c-subunit involves rotation by 36°, the difference between these sub-state structures also reveals information relevant to the 11° synthase-direction steps. Similar 11° and 25° rotational sub-states have also been observed with ADP-inhibited *Bt*F_1_F_O_ and *Ms*F_1_F_O_ (Zhou et al., 2015; Guo et al., 2021). In the latter, the binding of bedaquiline stabilizes a rotational sub-state that is either 25° CW or 8° CCW from the equivalent rotational state in the absence of the drug (Guo et al., 2021). The rotational position of the c-ring in cryo-EM structures of *Saccharomyces cerevisiae Sc*F_1_F_O_ is also changed by ~9° when the inhibitor oligomycin is bound to F_O_ (Srivastava et al., 2018).

#### Alternating two-step mechanism of F_O_-dependent synthase direction c-ring rotation

**Figure 14:**
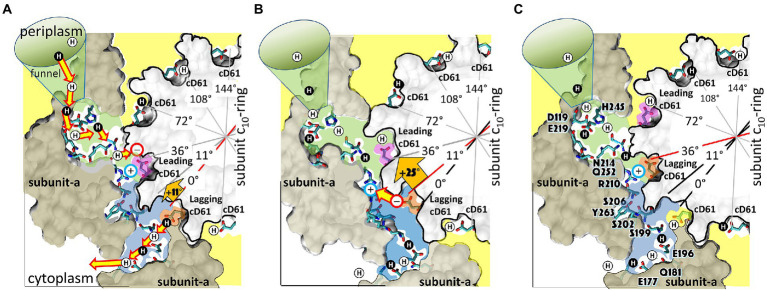
Alternating 11° and 25° sub-steps that power F_O_ c-ring ATP synthase direction rotation. **(A)** The pH-dependent 11° sub-step occurs when H^+^ transfer from aN214/aQ25-bound water to the unprotonated leading cD61-carboxyl (pink), and from the protonated lagging cD61-carboxyl (orange) to aS199/aE196-bound water. Upon displacement from aR210 by protonation, leading cD61 adopts the closed conformation to enable rotation into the lipid bilayer (yellow). Due to coherent H^+^ movement in the Grotthuss column, each H^+^ entering the input channel (green) from the funnel causes a H^+^ to exit the output channel (blue) to the cytoplasm. Rotation occurs when lagging cD61 is deprotonated because the negatively charged carboxyl moves in response to the decrease in hydrophobicity from the lipid bilayer to the water-containing vestibule (blue), and from the electrostatic attraction to aR210. This decreases the distance between the lagging cD61 carboxyl and the aR210-guanidinium from ~11.5 Å to ~7.5 Å. **(B)** The 25° sub-step occurs primarily from the electrostatic interaction between the lagging cD61 carboxy (orange) and the aR210 guanidinium. **(C)** Electrostatic attraction decreases the distance between orange cD61 and aR210 from ~7.5 Å to ~3.5 Å to complete a 36° stepwise c-ring rotation, which positions the orange cD61 to become the leading carboxyl for the next pH-dependent 11° sub-step. *E. coli* F_1_F_O_ cryo-EM structures of rotary sub-states pdb-IDs 5OQS **(A,C)**, and 5OQR **(B)** are shown as cross-sections of F_O_ with hydrophobic resides of subunit-a (brown) and the c-ring (gray) along the plane defined by cD61 groups as viewed from the periplasm. Protons are alternately colored black and white to show the progression of proton transfer events. This figure was modified from Yanagisawa and Frasch, (2021).

The alternating two-step mechanism to power synthase-direction c-ring rotation (Figure 14) is based on single-molecule rotation experiments of *Ec*F_1_F_O_ (Ishmukhametov et al., 2010; Martin et al., 2015), the effects of mutations of residues involved in F_O_ translocation on the pH-dependence of rotation (Yanagisawa and Frasch, 2017, 2021), and available F_1_F_O_ structures including *Ec*F_1_F_O_ sub-state structures 6OQR (Figure 14B) and 6OQS (Figure 14C) that differ due to rotation of the c_10_-ring relative to subunit-a by 25° (Sobti et al., 2020). Since rotation by a single c-subunit in the c_10_-ring is 36°, an 11° sub-step is required to return the c-ring from that of 6OQS (Figure 14A) to its original position of 6OQR (Figure 14B). This is consistent with the 11° synthase-direction steps that occur every 36° in the *Ec*F_1_F_O_ single-molecule studies (Yanagisawa and Frasch, 2017, 2021).

Several structural features of the *Ec*F_O_ motor, which are common to F-type ATP synthase, are relevant to the Alternating Two-step Mechanism of proton translocation-dependent c-ring rotation during ATP synthesis (Figures 13A, 14). Each c-subunit contains a carboxyl group that transports individual protons from the input channel to the output channel. Only two c-subunits in the ring contact subunit-a at a time, which are designated the leading (pink) and lagging (orange) for CW synthase-direction rotation. Leading and lagging carboxyls (subunit-cD61 in *E. coli*) are protonated and deprotonated by the respective input and output channels. The remaining carboxyl groups are protonated and rotate through the hydrophobic core of the membrane (yellow) until they reach the output channel. Positively charged aR210 is positioned adjacent to residues aN214 and aQ252 (green), which protonate the leading c-ring carboxyl group such that it can rotate away from subunit-a and into the hydrophobic lipid membrane. An aqueous vestibule (blue) that extends from aR210 to the initial output channel residues, aE196 and aS199, is formed by the interface between subunit-a and the c-ring. The vestibule is lined by subunit-a polar residues above and below the plane of rotation of the c-ring carboxyl groups, which aligns with aN214/aQ252 and aE196/aS199. Residues that provide a possible path for the output channel from aE196 to the cytoplasm include aQ181, aE177, and the subunit-a C-terminal carboxyl, which span this distance a ~ 4 Å intervals, consistent with that needed to stabilize a Grotthuss water channel. However, these are not conserved in several species.

**STEP-1.** *The 11° sub-step is powered by proton translocation to and from the subunit-a output and input channels from the lagging and leading cD61 carboxyls of the c-ring, respectively* (Figures 14A,B). In the 6OQS structure (Figure 14A), the lagging cD61 (orange) is 3.5 Å from the aS199 which enables proton transfer to aS199 and aE196 *via* bound water. The leading cD61 (pink) is 3.8 Å from the aR210-guanidinium, consistent with intervening water. This cD61 is also proximal to aN214 and aQ252, which positions it for protonation from the input channel *via* bound water. The pH-dependent 11° sub-step occurs upon proton transfer from water bound to aN214 and aQ252 to the leading cD61, and by proton transfer from the lagging cD61 to aS199 and aE196. Protonation from the input channel is vital to displace the unprotonated cD61 from the aR210 guanidinium, which eliminates the charge of cD61 to enable rotation into the hydrophobic lipid bilayer.

***STEP-2.** The 25° sub-step is powered by electrostatic attraction of the lagging cD61 carboxyl deprotonated in Step-1 to the aR210 guanidinium group* (Figures 14B,C). Deprotonation of the lagging cD61 by aE196/aS199 in Step-1 decreases the distance between this carboxyl group and the aR210 guanidinium from ~11.5 Å to ~7.5 Å (Figure 14C). These distances are inconsistent with the long-held belief that the role of aR210 is to displace the proton from the lagging cD61. Instead, the electrostatic attraction between the now negatively charged lagging cD61 and aR210 enables the 25° sub-step, which decreases the distance between them from 7.5 to 3.8 Å.

The occurrence of TDs may appear to be stochastic, but their occurrence depends on the kinetics and the energetics of the system. Slowing the angular velocity of the F_1_ ATPase-driven power stroke increases TD occurrence at pH 8.0 (a suboptimal pH), indicating that the ability to form a TD depends on the rate that an interaction can form between subunit-a and each c-subunit relative to the angular velocity of F_1_-ATPase-driven rotation. Evidence supports the hypothesis that the energy for F_1_-ATPase power strokes is derived from ATP binding-dependent closure of the β-subunit lever domain as it acts upon subunit-γ, which is initiated at ~36° after the catalytic dwell in *Ec*F_1_ (Martin et al., 2014). Based on the K_D_ of ATP at 36° measured in *Gs*F_1_, the energy available for the power stroke from ATP binding is ~13.5 k_B_T (Adachi et al., 2012). Consequently, the F_O_ motor must have at least 13.5 k_B_T available to cause a TD.

Two single-molecule observations indicate that the energy available to cause a TD are close to 13.5 k_B_T. *First*, TDs that occur early during F_1_-ATPase-dependent rotation mostly lack oscillations consistent with power strokes. Thus, the energy available to form the TD is clearly greater than that which causes ATPase-dependent rotation. However, oscillations are observed much more often during TDs that occur late in the F_1−_ATPase power stroke, which is consistent with a Brownian ratchet where the energy to drive F_O_-dependent CW rotation is close to that available by F_1_ to drive CCW rotation. *Second*, the low, medium, and high efficiencies of TD formation were attributed to torsional strain resulting from the asymmetry between 36° c_10_-ring stepping, and the 120° F_1_ power stroke. The torsional energy stored from the ±14° mismatch between F_1_ and F_O_ amounts to only 0.4 k_B_T (Yanagisawa and Frasch, 2021) calculated using the spring constant measured for *Ec*F_1_F_O_ (Sielaff et al., 2008). The fact that such a small difference in energy can significantly alter the efficiency of TD formation supports the conclusion that the energy used by F_O_ to generate TDs is close to that of the F_1_-ATPase power stroke.

The difference in pKa values between input and output channels measured by our single-molecule studies can provide as much as 4.4 k_B_T that can be used to rotate the c-ring in the ATP synthase-direction (Yanagisawa and Frasch, 2021). The desolvation energy of the negatively charged carboxyl group of the lagging cD61 upon deprotonation generates 5.9 k_B_T of energy (White and Whimley, 1999) because that charged moiety is excluded from the lipid bilayer into the aqueous vestibule. Conversely, the energy penalty of 0.8 k_B_T needed to insert the leading protonated cD61 carboxyl into the lipid bilayer (White and Whimley, 1999) is avoided by its conversion to the closed and locked conformation in the c-ring (Pogoryelov et al., 2010). The energy of the electrostatic attraction of aR210 to the unprotonated lagging cD61 is highly dependent upon the distance between the charges and the dielectric constant, which is a measure of the hydrophobicity of the environment that ranges from 2 (lipid bilayer) to 80 (aqueous solvent).

If these groups were both directly in the lipid bilayer separated by 11.5 Å, their electrostatic interaction would be as much as 38.1 k_B_T. Although we do not yet know how wet the vestibule is, a dielectric constant of 13 and an 11.5 Å aR210-cD61 distance results in 3.8 k_B_T, which when summed with the other energy sources totals 14.1 k_B_T without input of torsional energy. Since F_1_-ATPase rotation from the catalytic dwell to the point that ATP binds is powered by no more than 4 k_B_T (Martin et al., 2018), this explains why synthase-direction rotation near this rotational position typically has power stroke characteristics.

After the 25° rotation step when the unprotonated cD61-aR210 distance is 3.8 Å, the electrostatic energy is 11.4 k_B_T or 73.8 k_B_T when the dielectric constant is 13 or 2, respectively (Yanagisawa and Frasch, 2021). Thus, in a strictly hydrophobic environment the electrostatic interaction would be far too strong for any rotation to occur. For sustained CW rotation during ATP synthesis, the sum of the energy inputs from the pKa differences, desolvation energies, and pmf must be enough to displace cD61-aR210 when separated by 3.8 Å.

Recently, all-atom free energy calculations of *Sc*F_O_ found independent evidence of the 11° sub-step (Marciniak et al., 2022), confirmed that the differences in pKa values bias c-ring rotation in the synthase direction, and revealed similar energetic contributions from desolvation energies and electrostatic interactions to that observed from our single-molecule studies (Yanagisawa and Frasch, 2021). In addition, Marciniak et al. (2022) found that the yeast equivalent of aR210 (aR176) dictates the direction of rotation, controls the protonation state of the proton-release site, and separates the two input and output channels. As a result, this arginine is necessary to avoid slippage between proton flux and the mechanical output to guarantee highly efficient energy conversion.

Substate structures of ScF_1_F_O_ were recently solved by cryo-EM (Guo and Rubinstein, 2022) in which each of the three 120° F_1_ rotational contained substates in which F_1_ was either in the catalytic dwell position or the ATP-binding position, each of which was further distinguished by different F_O_ substates that differed by rotational single c-subunit stepping of its c_10_-ring. Most of the torsional strain that resulted from these single c-subunit steps was taken up by the compliance of the peripheral stalk. The number of rotational c-subunit steps observed differed among the three F_1_ states consistent with the low, medium, and high efficiency c-subunit stepping observed in the single-molecule studies (Yanagisawa and Frasch, 2017, 2021). Comparing the single-molecule studies and the new cryo-EM results will lead to a greater understanding of the molecular mechanism of ATP synthesis. More work is required to quantify the energetics of these sub-steps during ATP synthesis because, when understood in the context of steady-state pmf values, and the dissociation constants of ATP, ADP, and Pi versus rotary position, these energy contributions will determine the non-equilibrium ATP/ADP•Pi concentration ratio that can be maintained by F_1_F_O_ at steady-state *in vivo*.

## Frontiers of F_1_F_O_ mechanisms and nanotechnology applications of molecular motors

Interest in using molecular motors including F_1_ for nanotechnology applications began soon after single-molecule techniques were developed that enabled the visualization of molecular motor movements (Spetzler et al., 2007; York et al., 2008). This also sparked an interest in the design of synthetic molecular motors inspired by the F_1_-ATPase that has continued to grow. Designing a synthetic motor that rotates unilaterally by 360° by consuming a chemical fuel like that of F_1_ remains a challenge and is still in its infancy (Mo et al., 2022). However, the possibilities inherent in the development of these new technologies underscore the importance of understanding the mechanism of the F-type ATP synthase at a detailed level.

The significant progress in understanding the molecular mechanisms of both the F_1_ and F_O_ complexes has resulted from the development of new approaches to study the structure and function of these motors, of which several are described here. However, several important questions remain. A major unresolved question concerns the molecular basis for the differences among species in the rotary positions when the F_1_-ATPase binds ATP, hydrolyzes it, and when products are released. This is a conundrum since the sequence homologies of the αβ-heterodimers are so high, particularly among the residues that contribute to the catalytic sites. Although twisting of the subunit-γ coiled-coil occurs and contributes significantly to the ATPase mechanism of purified F_1_, the extent to which subunit-γ twisting contributes to the mechanism of ATP synthesis catalyzed by F_1_F_O_ remains to be determined.

The reasons for the >2-fold species variations in c-ring sizes of F_O_ are also major unresolved questions. From a bioenergetics perspective, the energy requirements of 8 H^+^ and 15 H^+^ to make three ATPs in mitochondria and chloroplasts is significant and the underlying reasons for these differences are unexplained. This difference also raises questions about the molecular mechanism of F_O_ because c_8_-rings and c_15_-rings rotate 45° and 24°, respectively, with each proton translocated across the membrane. The residues that comprise the subunit-a input channel are mostly characterized. However, except for aE196 and aS199, residues that participate in the output channel are not well conserved and remain to be identified. Although there is good evidence that protons are transported across the membrane *via* a Grotthuss mechanism, input and output channels of ATP synthases that transport Na^+^ must have specific adaptations that are not yet understood.

## Author contributions

WF, ZB, and SY wrote the manuscript. WF conceived the work and obtained funding. All authors contributed to the article and approved the submitted version.

## Funding

This work was funded by NIH R01GM097510 to WF, and from NSF-BII 2119963.

## Conflict of interest

The authors declare that the research was conducted in the absence of any commercial or financial relationships that could be construed as a potential conflict of interest.

## Publisher’s note

All claims expressed in this article are solely those of the authors and do not necessarily represent those of their affiliated organizations, or those of the publisher, the editors and the reviewers. Any product that may be evaluated in this article, or claim that may be made by its manufacturer, is not guaranteed or endorsed by the publisher.
